# Zfp462 Is a Key Mediator of Osteoblast Differentiation and Might Contribute to Age‐Related Bone Loss

**DOI:** 10.1111/acel.70476

**Published:** 2026-04-16

**Authors:** Jin‐Man Kim, Ho Kyoung Kim, Sung‐Ah Moon, Yewon Kim, Seung‐Hoon Lee, Jiyoung Yu, Seung Hun Lee, Woo Chan Son, Jung‐Eun Kim, In‐Jeoung Baek, Young‐Bum Kim, Jung‐Min Koh

**Affiliations:** ^1^ Asan Medical Center Asan Institute for Life Sciences Seoul Republic of Korea; ^2^ AMIST, Asan Medical Center University of Ulsan College of Medicine Seoul Republic of Korea; ^3^ Department of Molecular Medicine, CMRI, School of Medicine Kyungpook National, University Daegu Republic of Korea; ^4^ Convergence Medicine Research Center Asan Institute for Life Sciences Seoul Republic of Korea; ^5^ Division of Endocrinology and Metabolism, Asan Medical Center University of Ulsan College of Medicine Seoul Republic of Korea; ^6^ Department of Pathology, Asan Medical Center University of Ulsan College of Medicine Seoul Republic of Korea; ^7^ Department of Cell and Genetic Engineering, Asan Medical Center University of Ulsan College of Medicine Seoul Republic of Korea; ^8^ Division of Endocrinology, Diabetes, and Metabolism Beth Israel Deaconess Medical Center and Harvard Medical School Boston Massachusetts USA

**Keywords:** Moz, osteoblasts, Runx2, senile osteoporosis, Zfp462

## Abstract

Senile osteoporosis is characterized by a progressive decline in bone formation. Our study identifies Zfp462/ZNF462 as a novel regulator of osteoblast differentiation, providing new mechanistic insights into the aging‐related change in bone formation. Here, we demonstrate that ZNF462, MOZ, and RUNX2 physically interact with each other and promote osteoblastic bone formation by increasing RUNX2 activity and histone H3 acetylation. Importantly, we reveal that aging decreases ZNF462 expression in bone cells, a process linked to reduced occupancy of the histone variant H2A.Z at the *ZNF462* locus, leading to lower transcriptional activator histone H3K4 trimethylation. The osteoblast‐specific *Zfp462* deficiency in mice results in decreased bone mass and strength, primarily due to impaired osteoblast function. By uncovering a previously unknown ZNF462‐MOZ‐RUNX2 axis, this work provides a molecular basis for understanding the development of senile osteoporosis. Thus, targeting ZNF462 or MOZ could offer a new strategy to restore bone formation in aging populations.

## Graphical abstract

Osteoblast‐specific *Zfp462* deficiency reduces bone mass and strength, as well as the number of osteocalcin‐positive cells. Zfp462 forms a transcriptional complex with Moz and Runx2 to promote histone H3 acetylation and activate osteogenic gene expression. Aging reduces H2A.Z occupancy at the *Zfp462* locus, leading to decreased H3K4 trimethylation and transcriptional repression of *Zfp462* in bone cells.

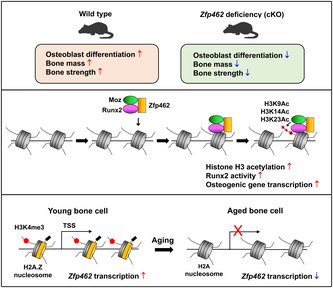

## 1 Introduction

Given the aging of the world's population, senile osteoporosis has become a significant public health concern (Aspray and Hill 2019). This condition is more complex than postmenopausal osteoporosis, which primarily results from estrogen deficiency that greatly increases bone resorption along with a more modest increase in bone formation. By contrast, senile osteoporosis is characterized by both increased bone resorption and diminished bone formation (Demontiero et al. 2012). The underlying causes of this decline in bone formation remain poorly understood, although several factors, including secondary hyperparathyroidism and cellular senescence (Boonen et al. 1995; Foger‐Samwald et al. 2022), have been proposed. In addition, the mechanisms that drive age‐related decline in bone formation likely involve Runt‐related transcription factor 2 (Runx2), which is a well‐known transcription factor that plays an essential role in early osteoblast differentiation (Ducy et al. 1997). Runx2 interacts with co‐regulator proteins such as Osterix/Sp7, TWIST, STAT1, SCHNURRI3, and zinc‐finger protein Zfp521 (Corrado et al. 2020; Hojo 2023), which collectively influence chromatin accessibility and transcription (Hojo et al. 2022). Aging is associated with reduced Runx2 expression, altered expression of its co‐regulators (Corrado et al. 2020), and changes in chromatin accessibility owing to histone modifications and histone variant incorporation (la Torre et al. 2023; Ma et al. 2021). However, how these elements interact to reduce osteoblast differentiation in aged humans/mice remains poorly understood.

Our previous genome‐wide association study (GWAS) and meta‐analysis with three replication cohorts (Hwang et al. 2013) identified zinc‐finger protein‐462 (*ZNF462*) as a potential contributor to senile osteoporosis. ZNF462 (Zfp462 in rodents) is a large protein (250 kDa) with 34 Cys2His2 zinc‐finger domains (Iuchi 2001). It is a transcription factor primarily known for its key roles in early embryonic development and neuronal differentiation (Laurent, Masse, Deschamps, et al. 2009; Laurent, Masse, Omilli, et al. 2009). Our GWAS data showed that a single‐nucleotide polymorphism (SNP) in *ZNF462* exhibited the strongest association with new vertebral osteoporotic fractures in the discovery cohort (*p* = 1.24 × 10^−5^) (Hwang et al. 2013). While this association was only marginally significant and technical issues prevented further SNP genotyping in the replication cohorts, this finding suggested a potential link between *ZNF462* and osteoporosis. Our interest in ZNF462 was further heightened when immunohistochemical staining analysis showed reduced ZNF462 expression in the bone marrow of patients with osteoporotic hip fractures compared to those without such fractures (described in the Results below). This finding is significant because (i) bone marrow is a key reservoir for osteoblasts and osteoclasts, and (ii) hip fractures, which mainly occur in much older adults, are a primary clinical marker of senile osteoporosis (Corrado et al. 2020). Additional support for a potential role of ZNF462 in bone metabolism comes from observations that (i) ZNF462/Zfp462 is highly expressed in the head and limbs of E12.5 mouse embryos, (ii) this expression decreases as mice mature into adulthood (Laurent et al. 2007), and (iii) age‐related skeletal changes are often initiated during juvenile growth deceleration (Lui et al. 2010). However, to date, there has been no explorations of the role of ZNF462 in bone metabolism, particularly regarding senile osteoporosis.

To address this, we conducted a series of clinical, genetic, and epigenetic analyses of ZNF462/Zfp462 in bone. Our findings suggested that ZNF462/Zfp462 interacts with and promotes the transcriptional activity of Runx2 in osteoblasts, which is also mediated by recruiting monocytic leukemic zinc finger (MOZ), a histone acetyltransferase that binds to both ZNF462/Zfp462 and Runx2 to modulate histones. Moreover, we show that ZNF462/Zfp462 expression in osteoblasts decreases with aging which is associated with reduced levels of the histone variant H2A.Z. These findings demonstrate that ZNF462/Zfp462 plays a critical role in the decreased bone formation that characterizes senile osteoporosis.

## 2 Materials and Methods

For more details on any of these sections, please see the Supporting Information.

### 2.1 Animals

All mice were on a C57BL/6 background. Most murine experiments were conducted on both male and female mice at 16 weeks of age. Calvarial osteoblasts were isolated from newborn mice. All animal protocols were approved by the Institutional Animal Care and Use Committee of Asan Institute of Life Sciences (Approval No. 2022‐12‐320). All animal studies followed ARRIVE guidelines 2.0.

### 2.2 Human Bone Specimens

Human bones used in the study were obtained from patients undergoing surgery, with informed consent obtained prior to specimen collection. This study was approved by the Institutional Review Board (IRB) of Asan Medical Center (Approval No. 2012‐0406).

### 2.3 Generation of *Zfp462*–Deficient Mice

Global *Zfp462* ^*−/−*^ knockout mice were generated on a C57BL/6 background using the Transcription Activator‐Like Effector Nucleases (TALEN) method (Sung et al. 2013) (Figure S1). For conditional deletion, *Zfp462* ^*f/f*^ mice were crossed with osteoblast‐specific *Col2.3‐Cre* mice (Riken, RBRC05603) to generate *Col2.3*; *Zfp462* ^*f/f*^ mice. All experiments on *Zfp462* global and conditional knockout mice were performed with littermate controls.

### 2.4 Statistical Analysis

All quantitative data are presented as mean ± SEM. Statistical comparisons between two groups were conducted using an unpaired two‐tailed Student's *t*‐test. For comparisons involving three or more groups, analysis of variance (ANOVA) was performed followed by either Tukey's or Sidak's post hoc test. A *p*‐value of < 0.05 was considered statistically significant.

## 3 Results

### 3.1 *Zfp462*/ *ZNF462* Deficiency Is Associated With Bone Loss, Possibly due to Impaired Bone Formation

**FIGURE 1:**
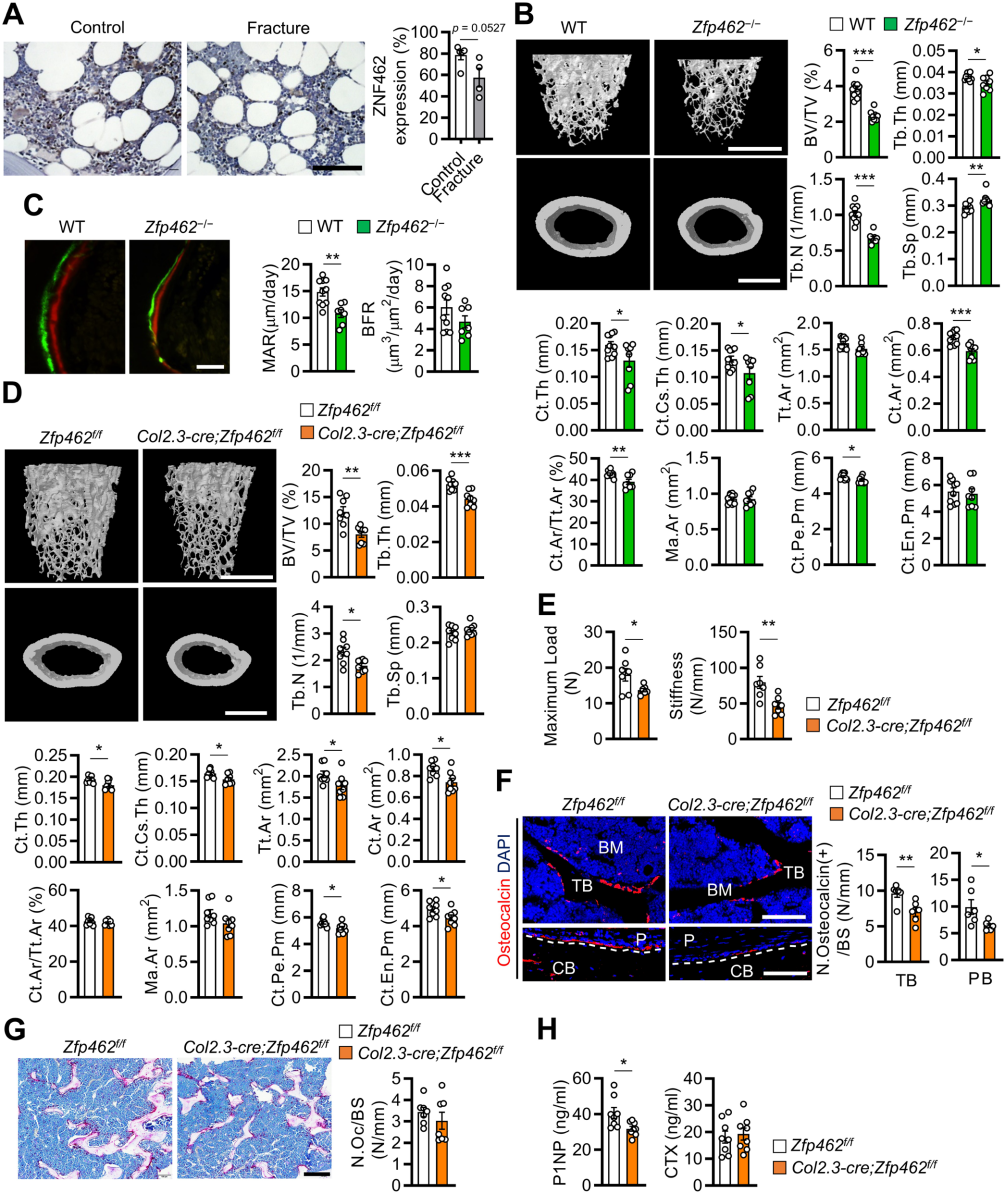
*Zfp462* deficiency in osteoblasts causes osteopenia and decreased bone formation. (A) Immunohistochemical staining of ZNF462 in proximal femurs of patients with (*n* = 4) or without (*n* = 5) osteoporotic hip fractures. Scale bar, 100 μm. (Right) Frequency of ZNF462‐positive cells. (B and C) Comparative analysis of femurs from 16‐week‐old female *Zfp462* ^*−/−*^ mice (*n* = 8) and WT littermates (*n* = 10). (B) Micro‐CT images and quantitation of trabecular and cortical bone variables. Scale bar, 1000 μm. BV/TV, trabecular bone volume per tissue volume; Tb.Th, trabecular thickness; Tb.N, trabecular number; Tb.Sp, trabecular spacing; Ct.Th, cortical thickness; Ct.Cs.Th, cortical cross‐sectional thickness; Tt.Ar, cortical tissue area; Ct.Ar, cortical area; Ma.Ar, marrow area; Ct.Pe.Pm, cortical periosteal perimeter; Ct.En.Pm, cortical endosteal perimeter. (C) Representative calcein and alizarin‐red double labeled trabecular bone in femurs from WT (*n* = 10) and *Zfp462* ^*−/−*^ mice (*n* = 7). Scale bar, 25 μm. (Right) Mineral apposition rate (MAR) and bone formation rate (BFR). (D–H) Male *Zfp462* ^*f/f*^ and *Col2.3‐cre*; *Zfp462* ^*f/f*^ mice were examined at 16 weeks of age. (D) Micro‐CT images of femurs (both *n* = 8). Scale bar, 1000 μm. (E) Maximum load (left) and stiffness (right) of tibiae, as measured via 3‐point bending analysis (both *n* = 7). (F) Representative images of osteocalcin‐immunostained (red) and DAPI‐stained (blue) femurs (both *n* = 6). Scale bar, 100 μm. BM, bone marrow; TB, trabecular bone; CB, cortical bone; P, periosteal. (Right) Osteocalcin‐positive cell numbers on trabecular bone (TB) and periosteal bone (PB) per bone surface. (G) Representative TRAP‐stained femur images (both *n* = 7). Scale bar, 200 μm. (Right) Osteoclast number per bone surface (N.Oc/BS). (H) Serum levels of procollagen‐1 N‐terminal peptide (P1NP) and collagen type‐I C‐terminal telopeptide (CTX) (both *n* = 8), as measured via ELISA. Data are shown as mean ± SEM. Statistical significance was determined using Student's unpaired two‐tailed *t*‐test. *p < 0.05, **p < 0.01, ***p < 0.001.

To determine whether ZNF462/Zfp462 is expressed in bone, we analyzed publicly available proteomics data and found its high expression in bone tissue (https://www.proteomicsdb.org/proteomicsdb/). Among the skeletal tissues examined, the highest expression was observed in the calvaria (Figure S2A). Moreover, BioGPS data revealed that murine osteoblasts and osteoclasts derived from bone marrow cells express high levels of *Zfp462* (http://www.biogps.org). We further observed that Zfp462 mRNA and protein expression steadily rose when bone marrow cells were induced to differentiate into osteoblasts or osteoclasts (Figure S2B–F). We conducted immunohistochemistry on proximal femurs from nine patients who underwent hip surgery owing to osteoporotic hip fracture (*n* = 4) or degenerative arthritis (*n* = 5). Patients with hip fractures were generally older and had lower bone mineral density than those with degenerative arthritis (Table S1). Notably, bone marrow from patients with hip fracture tended to express less ZNF462 compared with that of patients with degenerative arthritis (*p* = 0.0527) (Figure 1A). Thus, ZNF462 might play an important role in the physiology of bone cells associated with senile osteoporosis.

Whole‐body *Zfp462*‐deficient mice were generated. *Zfp462* ^*−/−*^ mice and their wild‐type (WT) littermates had similar body weights at 8–16 weeks, tibial length at 16 weeks, and organ weights at 4 weeks (Figure S3A–C). However, micro‐computed tomography (micro‐CT) revealed severe osteopenia in both male (Figure S3D) and female (Figure 1B) *Zfp462* ^*−/−*^ mice at 16 weeks. The trabecular bone volume/tissue volume (BV/TV) was significantly reduced in both sexes: male and female *Zfp462* ^*−/−*^ mice exhibited 71.4% ± 2.3% (Figure S3D) and 60.6% ± 2.6% (Figure 1B), respectively, compared with their WT littermates. Global deletion of Zfp462 appeared to result in more severe skeletal defects in female mice compared to male mice. Given prior reports indicating that estrogen receptor α activity is regulated by chromatin and transcriptional regulators (Schultz et al. 2010), and that ZNF462 has been implicated in the regulation of age at menarche (Perry et al. 2009), this sex‐specific difference may be attributable, at least in part, to the effects of estrogen on bone. Histomorphometry also confirmed this (Figure S3E). Moreover, many cortical bone variables were lower in aged male and female *Zfp462* ^*−/−*^ mice, although the affected variables differed: Ct.Pe.Pm was decreased in both; Tt.Ar, Ma.Ar, and Ct.En.Pm were reduced in males; and Ct.Th, Ct.Cs.Th, Ct.Ar, and Ct.Ar/Tt.Ar were decreased in females (Figure 1B, Figure S3D). We also found that *Zfp462* ^*−/−*^ mice had lower overall bone strength (Figure S3F). Importantly, 4‐week‐old *Zfp462* ^*−/−*^ mice exhibited no significant differences in lower trabecular or cortical bone variables compared with WT littermates (Figure S3G). This suggests that the bone loss in *Zfp462* ^*−/−*^ mice does not result from embryonic/neonatal abnormalities in bone development.

Histological analyses of femurs with tartrate resistant acid phosphatase (TRAP) and toluidine‐blue staining showed comparable osteoclast numbers but significantly fewer osteoblasts in *Zfp462* ^*−/−*^ mice at 16 weeks (Figure S4A). They also had significantly fewer osteocalcin‐positive cells (Figure S4B), reduced bone formation rates (Figure 1C), and lower circulating levels of bone formation markers, namely, alkaline phosphatase (ALP) activity and procollagen type‐1 N‐terminal propeptide (P1NP) (Figure S4C). By contrast, the levels of collagen type‐I C‐telopeptide (CTX), a bone resorption marker, remained unchanged (Figure S4C). Blood counts, the liver and kidney functions, and mineral concentrations in *Zfp462* ^*−/−*^ mice were normal (Table S2). Collectively, these findings suggested that whole‐body *Zfp462* deficiency reduces bone mass and strength, likely by inhibiting bone formation in vivo rather than by inducing systemic abnormalities or non‐bone diseases.

### 3.2 Osteoblast‐Specific *Zfp462* Deficiency Reduces Bone Mass Owing to Impaired Bone Formation

We generated mice that lacked *Zfp462* in only osteoblast lineage cells by mating *Zfp462* ^*f/f*^ mice with *Col2.3‐cre* mice that bore Cre recombinase driven by *2.3*‐kb type‐I *collagen* promoter (Figure S5A,B). Zfp462 expression was substantially reduced in differentiated osteoblasts, but not osteoclasts, from *Col2.3‐Cre*; *Zfp462* ^*f/f*^ mice (Figure S5C,D). At 16 weeks of age, male (Figure 1D) and female (Figure S5E) *Col2.3*; *Zfp462* ^*f/f*^ mice demonstrated trabecular BV/TV that was 65.5% ± 4.2% and 59.8% ± 7.4% of that in WT mice, respectively. Several other trabecular and cortical bone variables (Figure 1D, Figure S5E) along with bone strength (Figure 1E, Figure S5F) were reduced in both male and female *Col2.3*; *Zfp462* ^*f/f*^ mice, while calvarial cavity area was increased (Figure S5G). Histomorphometric analyses showed significantly fewer osteocalcin‐positive cells on the trabecular and periosteal bone surfaces in the mutant mice (Figure 1F), whereas osteoclast numbers were not affected (Figure 1G). Consistent with these findings, *Col2.3*; *Zfp462* ^*f/f*^ mice had lower serum P1NP levels but similar CTX levels (Figure 1H). These findings demonstrate that Zfp462 in osteoblasts is essential for bone formation, and its absence leads to osteopenia.

### 3.3 Zfp462/ZNF462 Increase Osteoblast Differentiation by Stimulating Runx2 Transcriptional Activity

**FIGURE 2:**
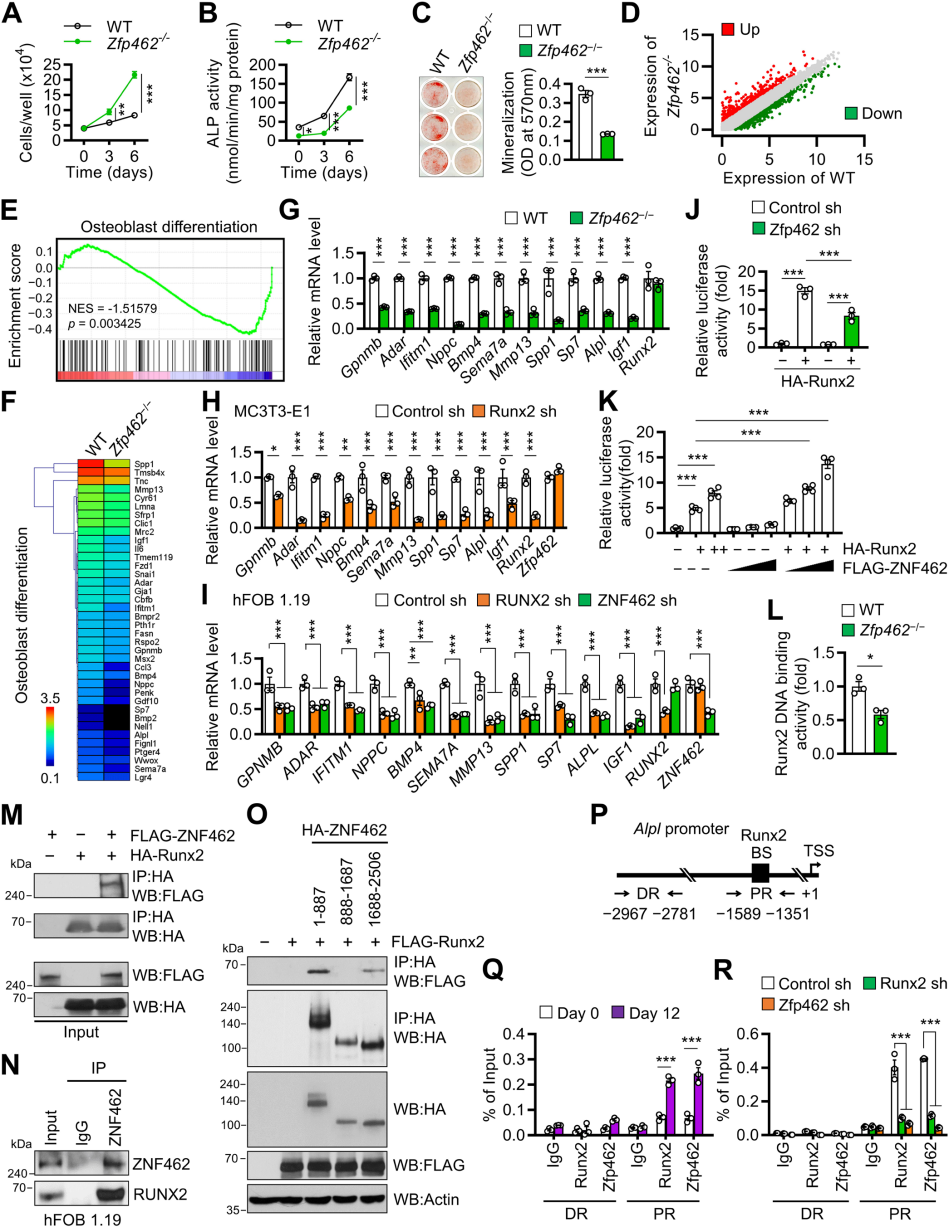
*Zfp462* deficiency decreases osteoblast differentiation by binding to Runx2 and inhibiting its transcriptional activity. (A) Calvarial osteoblast precursors derived from WT and *Zfp462* ^*−/−*^ mice were cultured for 6 days and counted at the indicated time points using a hemocytometer. (B and C) Calvarial osteoblast precursors from WT and *Zfp462* ^*−/−*^ mice were incubated in osteogenic medium. (B) After the indicated culture times, alkaline phosphatase (ALP) activity was determined. (C) After 12 days, osteoblasts were stained with alizarin red S and quantified (right). (D–F) RNA sequencing analysis of calvarial osteoblasts from WT and *Zfp462* ^*−/−*^ mice after 6‐day incubation of preosteoblasts in osteogenic medium. (D) Differentially expressed genes in *Zfp462* ^*−/−*^ osteoblasts (upregulated in red, downregulated in green). (E) Gene set enrichment analysis (GSEA) of osteoblast differentiation genes that were downregulated in *Zfp462* ^*−/−*^ osteoblasts. (F) Heatmap of osteoblast differentiation‐related genes in WT and *Zfp462* ^*−/−*^ osteoblasts. (G) GSEA data of WT and *Zfp462* ^*−/−*^ osteoblasts validated via qRT‐PCR analysis as described in (D–F). The expression of 11 osteoblast differentiation genes that were targeted by Zfp462 was quantified using qRT‐PCR and normalized to *18S*. *Runx2* expression was also assessed. (H and I) Effect of Runx2 or Zfp462 silencing on the expression of 11 Zfp462‐target genes in osteoblastic cell lines. (H) Murine MC3T3‐E1 cells treated with *Runx2* sh‐RNA and differentiated into osteoblasts for 6 days. (I) Human hFOB 1.19 cells transfected with *RUNX2* or *ZNF462* sh‐RNAs and differentiated into osteoblasts for 6 days. qRT‐PCR of the 11 Zfp462‐target genes was performed and normalized to *18S*. (J) Effect of Zfp462 silencing on Runx2 promoter activity. Murine MC3T3‐E1 cells were treated with *Zfp462* sh‐RNA and then transfected with 6 × OSE/luc reporter and 100 ng HA‐Runx2 expression vector. Reporter gene assay was performed 2 days after transfection. (K) Effect of *ZNF462* overexpression on Runx2 promoter activity. HEK293T cells were transfected with 6 × OSE/luc reporter, HA‐Runx2 (50 and 100 ng), and FLAG‐ZNF462 (125, 250, and 500 ng) expression vectors, after which the luciferase assay was performed. (L) DNA‐binding activity of Runx2 in nuclear extracts of calvarial osteoblasts from WT and *Zfp462* ^*−/−*^ mice. (M) HEK293T cells were co‐transfected with FLAG‐ZNF462 and HA‐Runx2 expression vectors. Immunoprecipitates generated post‐anti‐HA antibody incubation were analyzed via western blot using anti‐HA and anti‐FLAG antibodies. (N) Lysates of hFOB 1.19 cells were immunoprecipitated with an anti‐ZNF462 antibody and analyzed via western blot using anti‐ZNF462 and anti‐RUNX2 antibodies. (O) HEK293T cells were transfected with FLAG‐Runx2 and the indicated HA‐ZNF462 mutant expression vectors. Numbers indicate ZNF462 amino acid residues. Cell lysates were immunoprecipitated with an anti‐HA antibody, and the amount of Runx2 protein bound to the HA‐ZNF462 mutants was analyzed via western blot using anti‐FLAG and anti‐HA antibodies. The data presented in (M–O) are representative of three independent experiments. (P–R) Binding of Zfp462 and Runx2 to the *Alpl* locus in MC3T3‐E1 osteoblasts, as determined using chromatin‐immunoprecipitation (ChIP) analyses with anti‐Runx2 or anti‐Zfp462 antibodies. (P) Locations of distal regions (DR) and proximal regions (PR) in the *Alpl* locus that contain Runx2‐binding sequences (BS). Runx2‐BS were identified using the patch public 1.0 program in TRANSFAC Public 7.0 (http://gene‐regulation.com/). MC3T3‐E1 cells were induced to differentiate into osteoblasts by culturing in osteogenic media for 12 days. (Q) ChIP was conducted before or after osteogenesis using ChIP primers that included Runx2‐BS. (R) *Zfp462* or *Runx2* was silenced in MC3T3‐E1 cells, and ChIP was conducted with ChIP primers that included Runx2‐BS after incubation with osteogenic medium for the indicated times. Data are shown as mean ± SEM of three experiments except for (K) (*n* = 4). Statistical significance was determined using Student's unpaired two‐tailed *t*‐test (C, L), one‐way ANOVA followed by Tukey's test (J, K), or two‐way ANOVA followed by Sidak's post hoc test (A, B, G–I, Q, R). *p < 0.05, **p < 0.01, ***p < 0.001.

To elucidate how *Zfp462* promotes bone formation, we isolated osteoblast precursors from the calvaria of newborn *Zfp462* ^*−/−*^ and WT mice. *Zfp462* ^*−/−*^ preosteoblasts proliferated faster than WT preosteoblasts (Figure 2A). Calvarial precursors upon osteogenic induction displayed markedly lesser ALP activity (Figure 2B) and mineralization capacity (Figure 2C) than WT cells. Conversely, *Zfp462* knockout did not alter in vitro osteoclast formation and their bone resorption activity (Figure S6A,B). Thus, *Zfp462* promotes osteoblast differentiation.

RNA sequencing of calvarial *Zfp462* ^*−/−*^ and WT osteoblasts revealed that *Zfp462* knockout upregulated 436 genes and downregulated 575 genes by more than 2‐fold (Figure 2D). The altered genes were largely related to organ development and cell differentiation (Figure S6C). Gene‐set enrichment analysis (GSEA) scoring plot (Figure 2E) and heat‐map (Figure 2F) showed that *Zfp462* knockout also reduced the expression of many genes involved in osteoblast differentiation. Quantitative RT‐PCR (qRT‐PCR) confirmed that *Zfp462* knockout significantly suppressed the expression of 11 Zfp462‐target genes, including *Alpl*, *Sema7a*, and *Spp1* (Figure 2G).

Given that Runx2 is the master regulator of osteoblast differentiation (Bruderer et al. 2014), we investigated its interaction with *Zfp462*. *Zfp462* deletion did not affect *Runx2* expression in calvarial osteoblasts (Figure 2G, Figure S6D,E). Similarly, *Zfp462/ZNF462* knockdown in murine MC3T3‐E1 and human hFOB 1.19 osteoblastic cell lines did not alter *Runx2/RUNX2* expression (Figure S6F,G). Conversely, *Runx2/RUNX2* knockdown showed no effect on *Zfp462/ZNF462* expression in calvarial, MC3T3‐E1, or hFOB 1.19 osteoblasts (Figure S6F–H). However, knocking down *Runx2/RUNX2* in MC3T3‐E1 and hFOB 1.19 cells significantly suppressed the RNA expression of 11 osteoblast‐differentiation‐related Zfp462‐target genes (e.g., *Alpl/ALPL*, *Sema7a/SEMA7A*, and *Spp1/SPP1*) (Figure 2H,I). Notably, when we conducted a gene‐reporter assay with MC3T3‐E1 osteoblasts, we found that *Zfp462* knockdown suppressed Runx2 promoter activity (Figure 2J). Conversely, overexpressing ZNF462 in HA‐Runx2‐expressing HEK293T cells significantly increased Runx2 promoter activity (Figure 2K). As Runx2‐mediated activation of transcription requires binding Runx2 to its cognate DNA elements (Xiao et al. 1997), we next examined the effect of *Zfp462*/*ZNF462* knockdown on the DNA binding activity of Runx2/RUNX2 in calvarial, MC3T3‐E1, and hFOB 1.19 osteoblasts. Indeed, the knockdown blocked Runx2/RUNX2 binding to DNA in all three osteoblast types (Figure 2L, Figure S6I). Thus, Zfp462/ZNF462 is required to promote Runx2/RUNX2 activity without affecting its expression.

Immunoprecipitation analyses of the Runx2/ZNF462‐coexpressing HEK293T cells then showed that Runx2 physically interacted with ZNF462 (Figure 2M). Similarly, this was observed in hFOB 1.19 osteoblastic cells (Figure 2N). Analysis of HEK293T cells that co‐expressed Runx2 and deletion mutants of ZNF462 then demonstrated that Runx2 bound particularly strongly to the N‐terminal fragment of ZNF462 (Figure 2O). Moreover, chromatin immunoprecipitation (ChIP)‐qPCR assays with MC3T3‐E1 and hFOB 1.19 cells revealed that after osteogenesis, both Runx2/RUNX2 and Zfp462/ZNF462 bound at high levels to the proximal and not distal region of the Zfp462‐target genes *Alpl/ALPL*, *Sema7a/SEMA7A*, and *Spp1/SPP1* (Figure 2P,Q, Figure S7A,B). In addition, *Zfp462*/*ZNF462* knockdown suppressed Runx2/RUNX2 occupancy at these DNA regions (Figure 2R, Figure S7C,D). Interestingly, converse Runx2/RUNX2 knockdown suppressed *Zfp462*/*ZNF462* occupancy at the same DNA regions. Thus, Zfp462/ZNF462 binds to Runx2/RUNX2, and this binding allows both factors to further bind to the promoters of osteoblast‐differentiation‐related genes. This indicated that Zfp462/ZNF462 is a novel transcriptional cofactor of Runx2 that stimulates osteoblastic differentiation.

### 3.4 Moz Regulates Runx2 Transcriptional Activity in Cooperation With Zfp462/ZNF462

**FIGURE 3:**
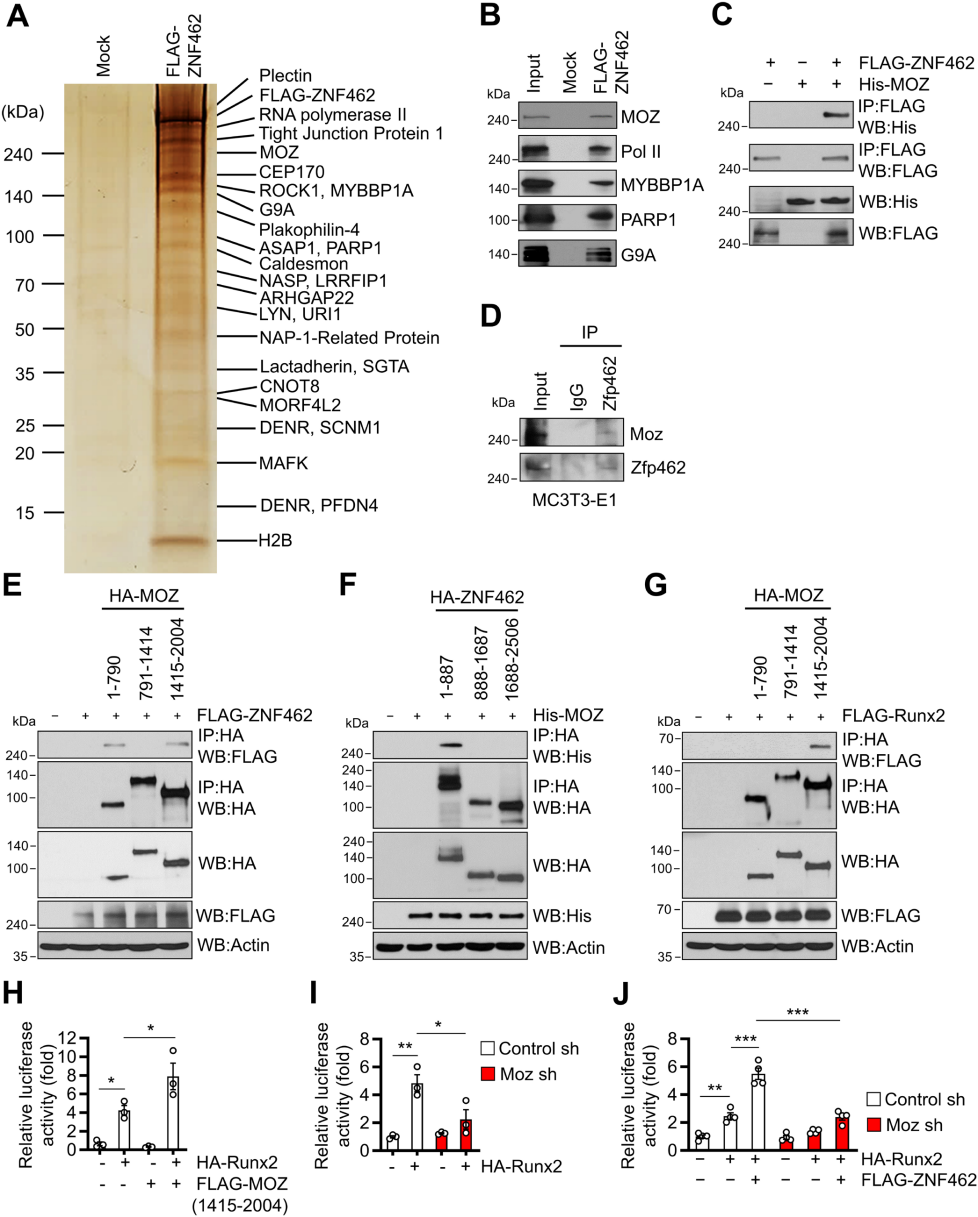
ZNF462 forms a complex with MOZ and Runx2. (A and B) Osteosarcoma U2OS cells (A) or HEK293T cells (B) were transfected with FLAG‐ZNF462 and then immunoprecipitated with an anti‐FLAG antibody. (A) ZNF462‐associated proteins were separated by 4%–20% SDS‐PAGE and analyzed using LC–MS/MS. (B) ZNF462‐associated proteins were separated using SDS‐PAGE and subjected to western blot analysis with the indicated antibodies. (C) HEK293T cells were co‐transfected with His‐MOZ and FLAG‐ZNF462, immunoprecipitated with anti‐FLAG antibody, and the levels of MOZ bound to ZNF462 were determined via western blot using anti‐HA and anti‐FLAG antibodies. (D) MC3T3‐E1 cells were immunoprecipitated with an anti‐Zfp462 antibody, and the levels of Moz bound to Zfp462 were determined via western blot using anti‐Moz and anti‐Zfp462 antibodies. (E) HEK293T cells were co‐transfected with FLAG‐ZNF462 and HA‐MOZ mutant expression vectors. Numbers indicate amino acid residues. Cell lysates were immunoprecipitated with anti‐HA antibody, and FLAG‐ZNF462‐bound proteins were analyzed using western blot using anti‐HA and anti‐FLAG antibodies. (F and G) Pull‐down assays with mutants in HEK293T cells were performed as described in (E) except using His‐MOZ and HA‐ZNF462 mutants (F) or FLAG‐Runx2 and HA‐MOZ mutants (G). The data presented in (B–G) are representative of three independent experiments. (H) HEK293T cells were transfected with 6 × OSE/luc reporter construct, 50 ng HA‐Runx2, and 250 ng FLAG‐MOZ (1415–2004) expression vectors and subjected to luciferase assay. (I and J) *Moz* was silenced in MC3T3‐E1 cells, and the cells were transfected with 6 × OSE/luc reporter together with 100 ng HA‐Runx2 (I) and/or 500 ng FLAG‐ZNF462 (J). Reporter gene assays were conducted 2 days post‐transfection. Data are shown as mean ± SEM of three experiments except for (J) (*n* = 4). Statistical analysis was conducted using one‐way ANOVA followed by Tukey's test. *p < 0.05, **p < 0.01, ***p < 0.001.

Yelagandula et al. (2023) recently showed that the ability of ZNF462/Zfp462 to regulate embryogenesis relies on its recruitment of the H3K9‐specific histone methyltransferase complex G9A/GLP. This complex binds to heterochromatin, which restricts transcription‐factor binding and thereby silences meso‐endodermal genes. Another zinc‐finger protein, Zfp521, can interact with histone deacetylase‐3 to antagonize Runx2 activity during osteoblast differentiation and maturation (Hesse et al. 2010), and the histone acetyltransferase MOZ interacts with Runx2 to enhance its transcriptional activity (Pelletier et al. 2002). Given this, we investigated whether other transcriptional regulators, particularly histone modulators, are involved in the interaction between ZNF462 and RUNX2 in osteoblasts. Osteosarcoma U2OS cells were transfected with FLAG‐ZNF462 and the immunoprecipitates generated using an anti‐FLAG antibody were analyzed through liquid chromatography–tandem mass spectrometry (LC–MS/MS). This analysis identified multiple proteins bound to ZNF462, including MOZ (Figure 3A). We further confirmed the binding of MOZ to ZNF462 in FLAG‐ZNF462‐expressing HEK293T cells via western blot analysis of the anti‐FLAG antibody immunoprecipitates (Figure 3B).

Given that MOZ interacts with Runx2 and improves its transcriptional activity (Pelletier et al. 2002), we further examined MOZ. Immunoprecipitation analyses showed that ZNF462/Zfp462 interacted with Moz/MOZ in both HEK293T cells (Figure 3C) and MC3T3‐E1 osteoblastic cells (Figure 3D). Moreover, mutation analyses in HEK293T cells indicated that (i) ZNF462 binds strongly to the N‐ and C‐terminal domains of MOZ (Figure 3E), (ii) similar to Runx2 (Figure 2O), MOZ interacts with the N‐terminal domain of ZNF462 (Figure 3F), and (iii) Runx2 interacts with the C‐terminal domain of MOZ (Figure 3G) akin to ZNF462 (Figure 3E). Reporter assays in HEK293T cells indicated that overexpressing the C‐terminal of MOZ significantly enhanced Runx2 promoter activity (Figure 3H). Conversely, *Moz* knockdown in MC3T3‐E1 osteoblasts repressed Runx2 promoter activity (Figure 3I), particularly when stimulated by ZNF462 overexpression (Figure 3J).

**FIGURE 4:**
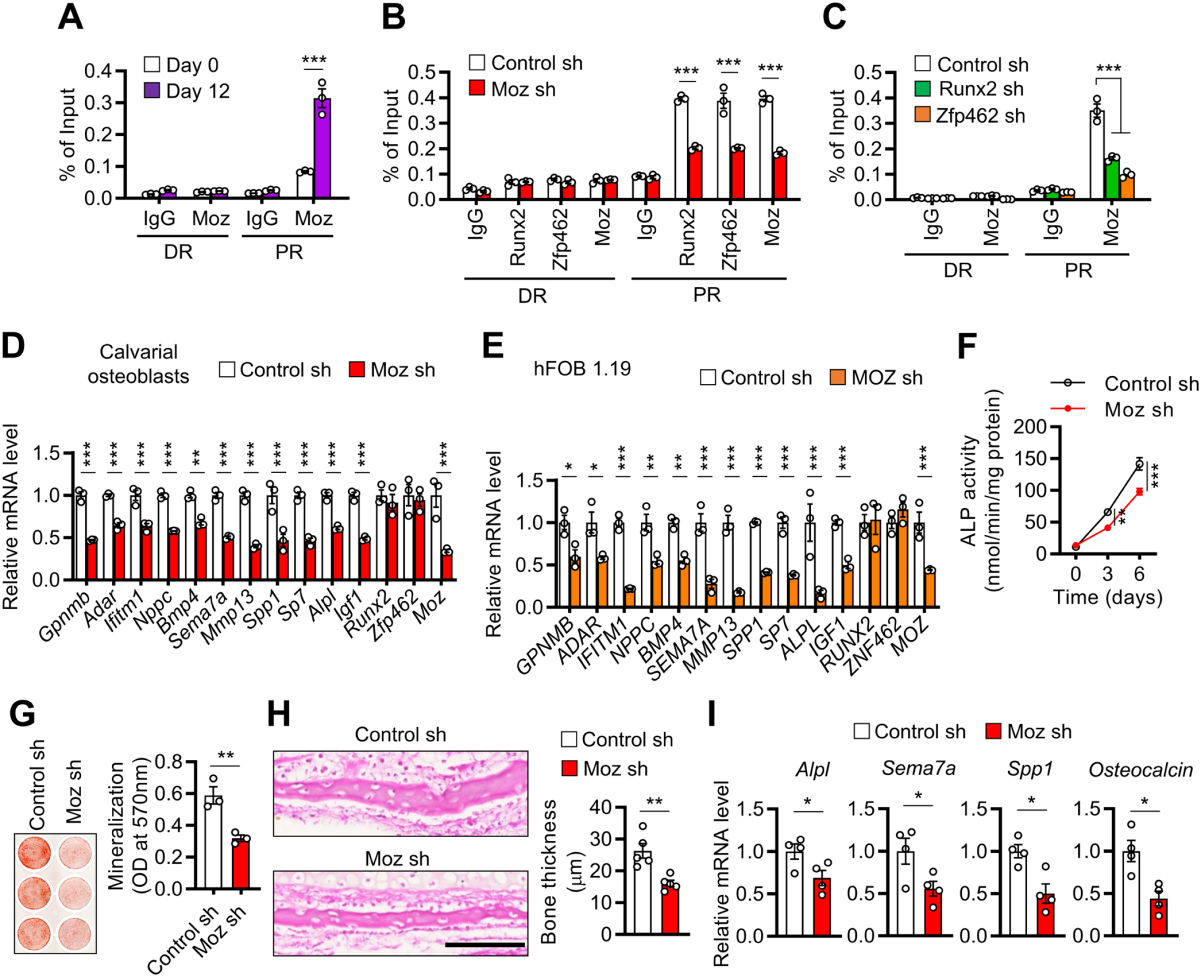
Moz promotes bone formation and bone mass. (A) MC3T3‐E1 cells were differentiated into osteoblasts using osteogenic medium for 12 days. Before and after differentiation, ChIP assays were performed at the distal regions (DR) and proximal regions (PR) of the *Alpl* locus using anti‐Moz antibody. (B) MC3T3‐E1 cells were treated with *Moz* sh‐RNA. ChIP assays were conducted at the *Alp1* locus with anti‐Runx2, anti‐Zfp462, and anti‐Moz antibodies. (C) MC3T3‐E1 cells were treated with *Runx2* or *Zfp462* sh‐RNAs, and ChIP assays were conducted at the *Alp1* locus using anti‐Moz antibody. (D and E) Calvarial (D) and hFOB 1.19 (E) osteoblast precursors were treated with *Moz*/*MOZ* sh‐RNA and differentiated into osteoblasts using osteogenic medium for 6 days. qRT‐PCR of 11 Zfp462‐target genes was performed. mRNA expression was normalized to *18S*. (F and G) Calvarial osteoblast precursors were treated with *Moz* sh‐RNA and incubated with osteogenic medium. (F) ALP activity was determined at the indicated time points. (G) After 12 days of incubation, calvarial osteoblasts were stained with alizarin red S. (Right) Alizarin red S was quantitated. (H and I) Whole calvariae were treated with *Moz* sh‐RNA and then incubated with osteogenic medium for 10 days. (H) Representative hematoxylin and eosin‐stained image. Scale bar, 100 μm. (Right) Relative bone thickness. (I) qRT‐PCR for *Alpl*, *Sema7a*, *Spp1*, and *Osteocalcin*. mRNA levels were normalized to *18S*. Data from three experiments (A–G) or four (I) or five (H) mice are shown as mean ± SEM. Statistical significance was determined by Student's unpaired two‐tailed *t*‐test (G–I) or two‐way ANOVA followed by Sidak's post hoc test (A–F). *p < 0.05, **p < 0.01, ***p < 0.001.

Similar to Runx2 and Zfp462 (Figure 2P–R, Figure S7), Moz/MOZ occupied the proximal regions of Zfp462‐target genes in MC3T3‐E1 and hFOB 1.19 osteoblasts (Figure 4A and Figure S8A,B). Notably, *Moz*/*MOZ* knockdown (Figure S8C,D) suppressed the recruitment of Moz/MOZ, Runx2/RUNX2, and Zfp462/ZNF462 to the target‐gene regions (Figure 4B, Figure S8E,F). Conversely, knocking down *Runx2*/*RUNX2* or *Zfp462*/*ZNF462* reduced Moz/MOZ recruitment at these regions (Figure 4C, Figure S8G,H). This suggests that Moz, Runx2, and Zfp462/ZNF462 may form a complex before binding to target genes.

*Moz/MOZ* knockdown in calvarial and hFOB 1.19 osteoblastic cells downregulated all Zfp462/ZNF462‐target genes without changing Runx2/RUNX2 and Zfp462/ZNF462 expression (Figure 4D,E, Figure S8C,D,I). *Moz* knockdown also reduced ALP activity and mineralization capacity in calvarial osteoblasts (Figure 4F,G). This finding was further verified ex vivo: treatment of calvarial explants with *Moz* short hairpin RNA (sh‐RNA) prevented the increase in calvaria thickness during the culture period, compared with control sh‐RNA‐treated calvariae (Figure 4H) and suppressed the expression of *Alpl*, *Sema7a*, *Spp1*, and *osteocalcin* in the calvariae (Figure 4I). These results suggested that Moz stimulates osteoblast differentiation and bone formation, likely by cooperating with Runx2 and Zfp462/ZNF462.

### 3.5 Moz Stimulates Osteoblast Differentiation by Acetylating Histone H3

**FIGURE 5:**
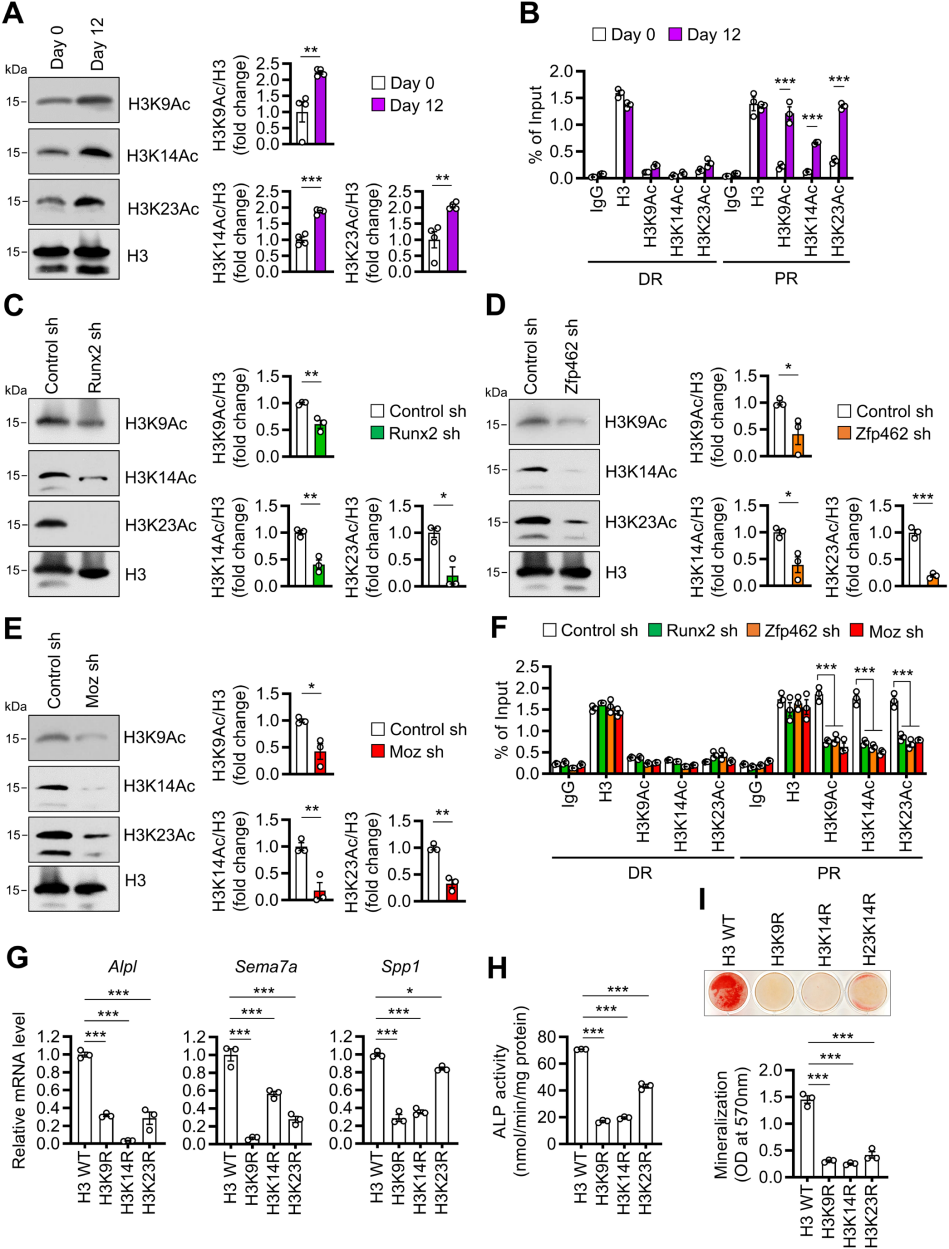
Zfp462‐Moz‐Runx2 complex regulates osteoblast differentiation in a histone H3 acetylation‐dependent manner. (A and B) MC3T3‐E1 cells were differentiated in osteogenic medium for 12 days. (A) Western blot was performed before and after differentiation with antibodies specific for the indicated histones. (Right) Band densities. (B) Before and after differentiation, ChIP assays at the distal regions (DR) and proximal regions (PR) of the *Alpl* locus were performed with anti‐H3K9Ac, anti‐H3K14Ac, anti‐H3K23Ac, and anti‐H3 antibodies. (C–E) MC3T3‐E1 cells were treated with *Runx2* (C), *Zfp462* (D), or *Moz* (E) sh‐RNAs, differentiated into osteoblasts using osteogenic medium for 12 days, and subjected to western blot with specific antibodies for the indicated histones. (Right) Band densities. (F) MC3T3‐E1 cells were treated with *Runx2*, *Zfp462*, or *Moz* sh‐RNAs, cultured in osteogenic medium for 12 days, and subjected to ChIP assays at the *Alpl* locus with anti‐H3K9Ac, anti‐H3K14Ac, anti‐H3K23Ac, and anti‐H3 antibodies. (G–I) MC3T3‐E1 cells were induced to stably overexpress WT H3 or H3 mutants, H3K9R, H3K14R, or H3K23R, and cultured in osteogenic medium. (G) After 6 days, relative mRNA levels of *Alpl*, *Sema7a*, and *Spp1* were quantified using qRT‐PCR. mRNA expression was normalized to *18S*. (H) After 12 days, ALP activity was measured. (I) Cells were stained with alizarin red S after 12 days. (Bottom) Alizarin red S was quantitated. Data are presented as mean ± SEM of three experiments except for (A) (*n* = 4). Statistical significance was determined using Student's unpaired two‐tailed *t*‐test (A, C–E), one‐way ANOVA followed by Tukey's test (G–I), or two‐way ANOVA followed by Sidak's post hoc test (B, F). *p < 0.05, **p < 0.01, ***p < 0.001.

Moz is a well‐known histone acetyltransferase (Perez‐Campo et al. 2013). We observed that differentiating MC3T3‐E1 and hFOB 1.19 cells into mature osteoblasts dramatically increased overall H3 acetylation while moderately increasing overall H4 acetylation (Figure S9A,B). This suggested that histone acetylation, particularly H3 acetylation, may be critical for osteoblast differentiation. MOZ is known to acetylate histone H3 lysine 9, 14, and 23 (H3K9, H3K14, and H3K23) (Huang et al. 2014; Kitabayashi et al. 2001; Voss et al. 2009). Indeed, their acetylated forms were more abundant in MC3T3‐E1 cells after osteoblastic differentiation (Figure 5A). Moreover, ChIP analysis revealed that H3K9Ac, H3K14Ac, and H3K23Ac were enriched in the proximal regions of Zfp462‐target genes in differentiated osteoblasts (Figure 5B, Figure S9C,D), the same regions where Runx2, Zfp462/ZNF462, and Moz are localized (Figures 2Q and 4A, Figures S7A,B and S8A,B,E,F). Knocking down *Runx2*, *Zfp462*, or *Moz* decreased overall H3K9Ac, H3K14Ac, and H3K23Ac levels (Figure 5C–E) and suppressed these acetylations at the proximal regions of Zfp462‐targeting genes in MC3T3‐E1 and hFOB 1.19 cells (Figure 5F, Figure S9E,F). Furthermore, when we mutated H3 at K9, K14, or K23 residues by arginine substitution, all mutations significantly decreased Zfp462‐targeting gene expression (Figure 5G), ALP activity (Figure 5H), and mineralization (Figure 5I). Collectively, these findings indicated that Moz stimulates osteoblast differentiation by binding to Runx2 and Zfp462, occupying the promoters of osteoblast differentiation genes, and increasing H3K9, H3K14, and H3K23 acetylation.

### 3.6 Aging Decreases Zfp462/ZNF462 Expression in Bone Cells by Reducing Histone Variant H2A.Z Levels

**FIGURE 6:**
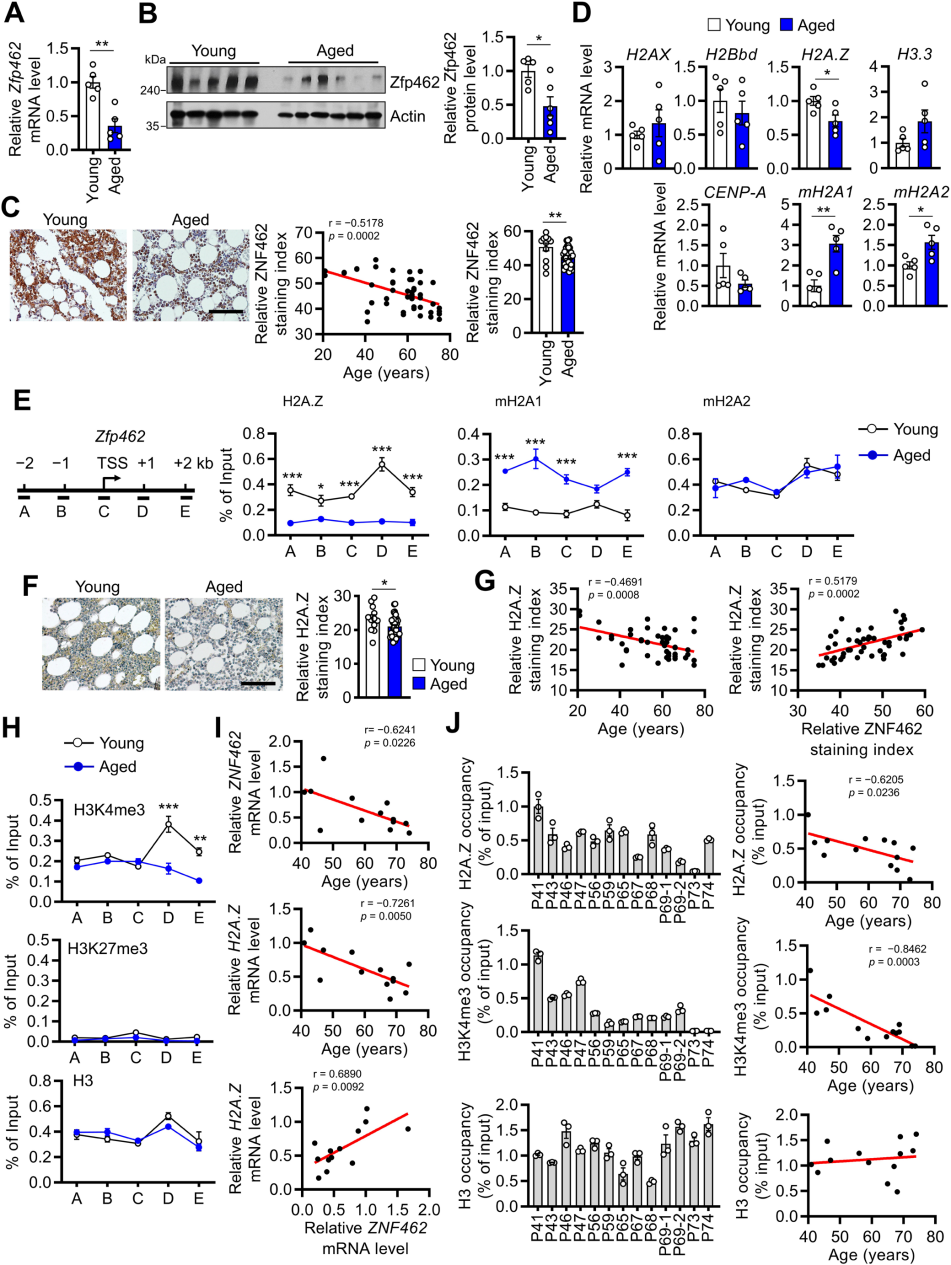
Aging decreases Zfp462/ZNF462 expression in osteoblasts by suppressing histone H2A.Z‐mediated histone H3K4‐trimethylation. (A and B) Calvariae were obtained from 3‐month‐old (young) and 20‐month‐old (aged) mice. The calvariae were subjected to qRT‐PCR (both *n* = 5) (A) and western blot of Zfp462 (young *n* = 5; aged *n* = 6) (B). (Right) Band densities. Actin was used as a loading control. (C) Immunohistochemical staining of ZNF462 in human tissue arrays from 24 subjects. The tissue was normal bone marrow in all cases. (Left) A representative staining. Scale bar, 100 μm. (Middle) Correlation between ZNF462 and age. (Right) ZNF462 expression in 6 young (< 50‐year‐old) and 18 aged (≥ 50‐year‐old) patients. (D and E) Calvariae were obtained from young and aged mice. (D) qRT‐PCR of the indicated histone variants was conducted (both *n* = 5). mRNA expression was normalized to *18S*. (E) ChIP assays were conducted at the *Zfp462* locus with anti‐H2A.Z, anti‐mH2A1, and anti‐mH2A2 antibodies (both *n* = 3). (Left) Approximate location of five amplicons at the *Zfp462* locus. (Right) H2A.Z, mH2A1, and mH2A2 levels in the *Zfp462* amplicons. (F and G) Immunohistochemical staining of H2A.Z in the human tissue arrays described in (C). (F) (Left) A representative staining. Scale bar, 100 μm. (Right) Quantification of H2A.Z expression. (G) Correlation between age and ZNF462 expression. (H) Calvariae were obtained from young and aged mice. ChIP assays were conducted at the *Zfp462* locus with anti‐H3K4me3, anti‐H3K27me3, and anti‐H3 antibodies (both *n* = 3). (I and J) Human bone marrow stromal cells (BMSCs) were obtained from 13 subjects without bone diseases and incubated with osteogenic media for 6 days. (I) qRT‐PCR of *ZNF462* and *H2A.Z* was performed. Correlations between *ZNF462* and *H2A.Z* and each gene with age. (J) ChIP assays were conducted at the region +1 kb upstream of the TSS of *ZNF462* gene with anti‐H2A.Z, anti‐H3K4me3, and anti‐H3 antibodies. (Left) Individual patient data are shown. Patients are arranged from youngest to oldest. (Right) Correlations of each histone type with age. Data are shown as mean ± SEM. Statistical significance was determined using Student's unpaired two‐tailed *t*‐test (A–D, F), two‐way ANOVA followed by Sidak's post hoc test (E, H), or Pearson's correlation analysis (C, G, I, J). *p < 0.05, **p < 0.01, ***p < 0.001.

Given that osteoporotic hip fracture, which is a marker of senile osteoporosis (Boonen et al. 2008), tended to be associated with lower ZNF462 levels in patient bone marrow (Figure 1A) and aging is linked to several transcriptomic changes (Perez‐Gomez et al. 2020), we hypothesized that Zfp462/ZNF462 expression may decline with age and contribute to senile osteoporosis. We obtained femurs from 3‐ and 20‐month‐old mice. The aged mice showed the same senile bone changes described previously (Beaucage et al. 2016; Shum et al. 2016). Particularly, compared with young mice, aged mice exhibited significantly lower bone‐mass variables but higher skeletal‐size variables, including total cross‐sectional area, cortical periosteal and endosteal perimeters, and marrow area (Figure S10A). Interestingly, the calvariae of aged mice showed significantly reduced Zfp462 mRNA and protein expression than those of young mice (Figure 6A,B). We examined human tissue arrays of healthy bone marrow from 24 subjects aged 21–75 years (Table S3). ZNF462 protein expression was inversely correlated with age (*r* = −0.5178, *p* = 0.0002), and bone marrows from subjects aged ≥ 50 years expressed significantly lesser ZNF462 than those aged < 50 years (Figure 6C). These indicated that aging is associated with reduced Zfp462/ZNF462 expression in bone.

To determine the mechanism by which aging decreases Zfp462/ZNF462 expression, we analyzed several epigenetic marks, as aging is associated with epigenetic changes, including DNA methylation and histone variant replacement, which regulate gene transcription (Pal and Tyler 2016). Bisulfite sequencing of the aged and young osteoblasts and osteoclasts did not differ in DNA methylation around the transcription start site (TSS) of *Zfp462* gene (Figure S10B). However, analysis of histone variants showed that aged calvariae expressed lower H2A.Z and higher macroH2A1 (mH2A1) and mH2A2 levels compared with young calvariae (Figure 6D). Moreover, ChIP assays demonstrated that aged calvariae had reduced H2A.Z levels at the *Zfp462* locus but higher mH2A1 levels, while mH2A2 levels remained unchanged (Figure 6E). In young mice, H2A.Z was highly enriched in the proximal coding region of *Zfp462* (region D). Conversely, mH2A1 levels in region D were reduced in aged mice (Figure 6E). Immunohistochemical analysis of the human bone marrow tissue arrays further supported these findings, as older subjects (≥ 50 years) exhibited lower H2A.Z levels than younger subjects (Figure 6F). H2A.Z expression showed negative and positive correlation with aging and ZNF462 expression, respectively (Figure 6G). mH2A1 expression was elevated in aged patients than younger patients and positively correlated with aging but not with ZNF462 expression (Figure S10C). These data suggested that aging decreases H2A.Z levels at the *Zfp462/ZNF462* locus in both murine and human bone.

H2A.Z and mH2A1 are known to associate with active and repressive histone marks, H3 lysine 4 tri‐methylation (H3K4me3) and H3 lysine 27 tri‐methylation (H3K27me3), respectively (Gaspar‐Maia et al. 2013; McHaourab et al. 2018). H3K4me3 distribution at the *Zfp462* gene in young and old murine calvariae (Figure 6H) was similar to that of H2A.Z (Figure 6E), whereas H3K27me3 was not detected in either group (Figure 6H). Among histone methyltransferases involved in H3K4me3 regulation (Wang and Helin 2025), expression of specific H3K4 methyltransferases, such as *Kmt2b* and *Kmt2c*, was altered in aged bones (Figure S10D), which may warrant further investigation into their contribution to age‐related alterations in H3K4me3 regulation. These findings suggested that aging leads to the loss of H2A.Z variant in the coding region of the *Zfp462/ZNF462* gene, which reduces H3K4me3 levels and may potentially diminish Zfp462/ZNF462 expression in bone.

To determine whether these aging‐related changes in ZNF462, histone variants, and H3K4me3 also occur in human bone cells, we obtained bone marrow stromal cells (BMSCs) from excised bones of 13 subjects aged 41–74 years (Table S4). BMSCs might contain osteoblast precursors (Zhou et al. 2022), and these patients lacked bone disease (Kim et al. 2003). We confirmed that *ZNF462* and *H2A.Z* expression were negatively correlated with aging in the BMSCs, whereas *ZNF462* expression was positively correlated with *H2A.Z* expression (Figure 6I, Figure S10E). By contrast, *mH2A1* levels did not correlate with either aging or *ZNF462* levels (Figure S10E,F). ChIP assays further revealed that in young patients, both H2A.Z and H3K4me3 peaked in the protein‐coding region of *ZNF462* locus (region D) (Figure S10G). Moreover, H2A.Z and H3K4me3 occupancy at the *ZNF462* locus were inversely correlated with aging, while mH2A1 occupancy did not (Figure 6J, Figure S10H). Collectively, aging decreases H2A.Z expression in bone cells including osteoblasts, which may then suppress histone H3K4 trimethylation, thereby decreasing expression of Zfp462/ZNF462.

## 4 Discussion

This study demonstrated that Zfp462/ZNF462 is a novel regulator of bone formation (Figure S11). In particular, Zfp462/ZNF462, Moz/MOZ, and the master osteoblast regulator Runx2/RUNX2 physically interact with each other and promote osteoblast differentiation and bone formation by increasing Runx2/RUNX2 activity and histone H3 acetylation. Thus, both Zfp462/ZNF462 and Moz/MOZ function as coactivators of Runx2/RUNX2. We confirmed that osteoblast‐specific *Zfp462* deficiency decreased bone mass and strength by impairing bone formation in vivo and that *Moz* knockdown also reduced bone formation ex vivo. We also found that Zfp462/ZNF462 may be involved in senile osteoporosis, because (i) it was expressed at lower levels in the bone marrow of patients with osteoporotic hip fracture, which is a hallmark clinical sign of senile osteoporosis (Boonen et al. 2008); and because (ii) its expression in human and murine bone cells drops with age. Aging reduced Zfp462/ZNF462 expression in bone cells by decreasing histone variant H2A.Z levels, and therefore H3K4me3 levels, at the *Zfp462/ZNF462* locus. These findings together suggest that amplifying Zfp462/ZNF462 or Moz/MOZ expression with targeted drugs could potentially improve bone formation in aged humans or prevent senile osteoporosis.

The ability of Zfp462/ZNF462 to interact directly with Runx2 and enhance its transcriptional activity is consistent with the behavior of other zinc‐finger proteins. In particular, proteins such as Gli2, TIEG1/KLF10, and Zfp521, all of which contain zinc‐finger motifs, have been shown to strengthen Runx2 binding to DNA and regulate Runx2 transcriptional activity (Hawse et al. 2011; Hesse et al. 2010; Shimoyama et al. 2007). Zfp462/ZNF462 shapes Runx2 transcriptional activity by recruiting Moz/MOZ, a transcription activator and histone acetyltransferase (Perez‐Campo et al. 2013), which is similar to the zinc‐finger protein Zfp521 that suppresses Runx2‐mediated gene expression in osteoblast maturation by recruiting histone deacetylase‐3, a transcription repressor (Hesse et al. 2010). Thus, Zfp462/ZNF462 behavior is broadly similar to that of other zinc‐finger proteins.

Our ChIP experiments using sh‐RNAs targeting *Zfp462/ZNF462*, *Moz/MOZ*, and *Runx2/RUNX2* confirmed that all three molecules bind to the proximal promoter regions of genes involved in osteoblast differentiation. Moreover, their physical interaction was necessary before they could bind to these DNA regions. As *Zfp462* knockout or *Moz* knockdown in murine calvarial osteoblast cells prevented their osteoblastic differentiation, Zfp462 and Moz are essential co‐activators of Runx2 in osteoblast development. Furthermore, we identified that these interactions are mediated by the N‐terminal domain of ZNF462 and the C‐terminal domain of MOZ, making these domains potential therapeutic targets for senile osteoporosis.

Senile osteoporosis arises from excessive bone resorption by osteoclasts and reduced bone formation by osteoblasts (Corrado et al. 2020). Various mechanisms have been reported to promote age‐related decline in bone formation, including diminished bone anabolic responses to mechanical loading, accumulation of senescent cells, increased osteoblast apoptosis, and decreased osteoblast differentiation. The latter is associated with age‐related alterations in the expression of Runx2 and other osteoblast differentiation factors such as FOXP, PPARγ, and Wnt10b (Corrado et al. 2020), as well as Runx2 co‐regulators (Shimoyama et al. 2007). Our findings align with these observations, showing that Zfp462/ZNF462 regulates Runx2/RUNX2 to stimulate bone formation, and its expression drops with age in both mouse and human bone cells.

Histone variant occupancy in nucleosomes changes with aging, and it can alter chromatin conformation and regulate transcriptional gene expression (Pal and Tyler 2016; Yi and Kim 2020). Our study suggests that the age‐related decrease in Zfp462/ZNF462 expression is driven by the progressive loss of histone variant H2A.Z at the *Zfp462*/*ZNF462* locus in bone cells. Particularly, we found that H2A.Z expression in murine and human bone tissues declines with age and positively correlates with ZNF462 expression. We demonstrated for the first time that Zfp462/ZNF462 and histone variant H2A.Z contribute to age‐related impairments in bone formation.

Loss of H2A.Z may reduce *Zfp462/ZNF462* expression by preventing the generation of active mark H3K4me3 at the *Zfp462/ZNF462* locus. Our findings indicated that aging not only reduces H2A.Z levels but also decreases H3K4me3 levels at this locus. Moreover, the distribution of H2A.Z and H3K4me3 within the *Zfp462/ZNF462* locus was identical in both younger and aged mice and humans. In particular, both showed an occupancy peak in the proximal coding region that disappeared with aging. Notably, this expression pattern of H2A.Z may be specific to bone cells or bone, as H2A.Z has been reported to increase with age in other cell types and tissues (Stefanelli et al. 2018). Therefore, H2A.Z incorporation at the *Zfp462*/*ZNF462* locus may enhance its gene expression in a histone H3K4me3‐dependent manner.

Aging is associated with trabecular bone mass loss, as shown by reduced trabeculae thickness and numbers and increased trabecular spacing (Corrado et al. 2020). Cortical bone thickness also decreases with age despite an increase in periosteal bone apposition (Corrado et al. 2020). In *Zfp462*/*ZNF462*‐deficient mice, most skeletal parameters showed changes consistent with aging; however, a reduction in the periosteal perimeter was observed, a finding not typically associated with age‐related skeletal changes. This suggests that Zfp462/ZNF462 may not be the only factor that determines age‐related bone changes and is unlikely to influence age‐related periosteal bone changes.

Weiss–Kruszka syndrome, recently described as a condition caused by ZNF462 haploinsufficiency, has been reported to include skeletal abnormalities in a subset of affected individuals (van der Laan et al. 2024). These findings include metopic ridging and craniosynostosis, as well as clinodactyly and short stature (Kruszka et al. 2019), features suggestive of impairments in both intramembranous and endochondral ossification. Although we did not detect significant differences in tibial length in *Zfp462* ^*−/−*^ mice, these clinical observations raise the possibility that Zfp462/ZNF462 may also play a role in skeletal development, thereby warranting further detailed and systematic investigation.

In conclusion, this study identifies Zfp462/ZNF462 and Moz as novel stimulators of bone formation that promote osteoblast differentiation by facilitating Runx2 activity. Moreover, aging suppresses Zfp462/ZNF462 expression in bone cells by reducing local levels of H2A.Z and H3K4me3. These findings thus elucidate a potential mechanism and molecular axis underlying senile osteoporosis and could potentially be targeted to ameliorate or prevent this increasingly prevalent condition.

## Author Contributions

Jung‐Min Koh conceived the idea and designed the experiments. Jin‐Man Kim, Ho Kyoung Kim, Sung‐Ah Moon, Yewon Kim, Seung‐Hoon Lee, Jiyoung Yu, Seung Hun Lee, Woo Chan Son, Jung‐Eun Kim, In‐Jeoung Baek, and Jung‐Min Koh conducted experiments and analyzed data. Jin‐Man Kim, Young‐Bum Kim, and Jung‐Min Koh wrote the manuscript.

## Funding

This work was supported by National Research Foundation of Korea, 2020R1A2C3006699, 2021R1F1A1057315. Korea Health Industry Development Institute, RS‐2024‐00438349, RS‐2024‐00401153.

## Conflicts of Interest

The authors declare no conflicts of interest.

## Supporting information


**Table S1:** Clinical characteristics of patients with and without osteoporotic hip fractures.
**Table S2:** Blood tests in male wild‐type (WT) and *Zfp462* ^*−/−*^ mice at 16 weeks of age.
**Table S3:** Demographic information of subjects who provided bone marrow for human tissue arrays that were subjected to immunohistochemistry.
**Table S4:** Clinical information of subjects who provided bone marrow stromal cells.
**Table S5:** Sequences of sh‐RNAs used in the study.
**Table S6:** Specific primer sequences for qRT‐PCR.
**Table S7:** Specific primer sequences for ChIP‐qPCR.
**Figure S1:** Generation of *Zfp462*‐deleted mice using the TALEN system.
**Figure S2:** Zfp462 expression in various bones and during osteoblast and osteoclast differentiation.
**Figure S3:** Organ weights and bone phenotype in male whole‐body *Zfp462*‐deficient mice at 4 and 16 weeks of age.
**Figure S4:** *Zfp462* deficiency decreases the number of osteoblasts, but not the number of osteoclasts.
**Figure S5:** Generation of *Col2.3‐cre*; *Zfp462* ^*f/f*^ mice and determination of bone mass and strength.
**Figure S6:** Effect of *Zfp462* deficiency on in vitro bone resorption and osteoblastic Runx2 expression.
**Figure S7:** ChIP analysis of Runx2/RUNX2 and Zfp462/ZNF462 binding at ZNF462/Zfp462‐target genes in osteoblasts.
**Figure S8:** ChIP analysis of Runx2/RUNX2, Zfp462/ZNF462, and Moz/MOZ binding at ZNF462/Zfp462‐target genes in osteoblasts.
**Figure S9:** ChIP analysis of histone H3 acetylation at ZNF462/Zfp462‐target genes in osteoblasts.
**Figure S10:** Histone variants in human bone marrow cells and their relationship with aging and *ZNF462* expression.
**Figure S11:** Proposed model of the mechanisms by which Zfp462‐Moz‐Runx2 complex modulates bone formation in an aging‐dependent manner.

## Data Availability

RNA sequencing data have been deposited in the Gene Expression Omnibus (GEO) under the accession code GSE263732.
