# Supplementary material for: Zfp462 Is a Key Mediator of Osteoblast Differentiation and Might Contribute to Age‐Related Bone Loss
Source: Aging Cell. 2026 Apr 16;25(4):e70476. doi: 10.1111/acel.70476 (PMC13086632; doi:10.1111/acel.70476)
Supplement: Supplementary file 1 — Table S1: Clinical characteristics of patients with and without osteoporotic hip fractures. Table S2: Blood tests in male wild‐type (WT) and Zfp462 −/− mice at 16 weeks of age. Table S3: Demographic information of subjects who provided bone marrow for human tissue arrays that were subjected to immunohistochemistry. Table S4: Clinical information of subjects who provided bone marrow stromal cells. Table S5: Sequences of sh‐RNAs used in the study. Table S6: Specific primer sequences for qRT‐PCR. Table S7: Specific primer sequences for ChIP‐qPCR. Figure S1: Generation of Zfp462‐deleted mice using the TALEN system. Figure S2: Zfp462 expression in various bones and during osteoblast and osteoclast differentiation. Figure S3: Organ weights and bone phenotype in male whole‐body Zfp462‐deficient mice at 4 and 16 weeks of age. Figure S4: Zfp462 deficiency decreases the number of osteoblasts, but not the number of osteoclasts. Figure S5: Generation of Col2.3‐cre; Zfp462 f/f mice and determination of bone mass and strength. Figure S6: Effect of Zfp462 deficiency on in vitro bone resorption and osteoblastic Runx2 expression. Figure S7: ChIP analysis of Runx2/RUNX2 and Zfp462/ZNF462 binding at ZNF462/Zfp462‐target genes in osteoblasts. Figure S8: ChIP analysis of Runx2/RUNX2, Zfp462/ZNF462, and Moz/MOZ binding at ZNF462/Zfp462‐target genes in osteoblasts. Figure S9: ChIP analysis of histone H3 acetylation at ZNF462/Zfp462‐target genes in osteoblasts. Figure S10: Histone variants in human bone marrow cells and their relationship with aging and ZNF462 expression. Figure S11: Proposed model of the mechanisms by which Zfp462‐Moz‐Runx2 complex modulates bone formation in an aging‐dependent manner. [file ACEL-25-e70476-s001.docx]

**Supplementary information**

**Zfp462 is a Key Mediator of Osteoblast Differentiation and Might Contribute to Age-Related Bone Loss**

Jin-Man Kim^1^, Ho Kyoung Kim^1^, Sung-Ah Moon^2^, Yewon Kim^1^, Seung-Hoon Lee^3^, Jiyoung Yu^4^, Seung Hun Lee^5^, Woo Chan Son^6^, Jung-Eun Kim^3^, In-Jeoung Baek^7^, Young-Bum Kim^8^, and Jung-Min Koh^5,*^

^1^Asan Institute for Life Sciences, Asan Medical Center, Seoul, 05505, Republic of Korea

^2^AMIST, Asan Medical Center, University of Ulsan College of Medicine, Seoul, 05505, Republic of Korea

^3^Department of Molecular Medicine, CMRI, School of Medicine, Kyungpook National, University, Daegu, 41944, Republic of Korea

^4^Convergence Medicine Research Center, Asan Institute for Life Sciences, Seoul, 05505, Republic of Korea

^5^Division of Endocrinology and Metabolism, Asan Medical Center, University of Ulsan College of Medicine, Seoul, 05505, Republic of Korea

^6^Department of Pathology, Asan Medical Center, University of Ulsan College of Medicine, Seoul, 05505, Republic of Korea

^7^Department of Cell and Genetic Engineering, Asan Medical Center, University of Ulsan College of Medicine, Seoul, 05505, Republic of Korea

^8^Division of Endocrinology, Diabetes, and Metabolism, Beth Israel Deaconess Medical Center and Harvard Medical School, Boston, MA, 02115, USA

^*^Correspondence: Jung-Min Koh, E-mail: [jmkoh@amc.seoul.kr](mailto:jmkoh@amc.seoul.kr)

**Supplementary Materials and Methods**

**Animals**

Mice were maintained under standard conditions with controlled temperature, humidity, and a 12-h light/dark cycle. Before euthanasia, all mice were intraperitoneally anesthetized with Zoletil (50 mg/kg) and xylazine (10 mg/kg). After euthanasia, organs were harvested for analyses.

**Generation of global *Zfp462^−/−^* mice**

Conventional whole-body *Zfp462^−/−^* knockout mice were generated on a C57BL/6 background using Transcription Activator-Like Effector Nucleases (TALENs). TALEN-mediated genome editing introduced a 14-bp deletion in exon 2 of the *Zfp462* gene, resulting in a frameshift mutation (Founder #3) (Figure S1A–D). Loss of Zfp462 expression was confirmed by the absence of *Zfp462* mRNA in major organs and lack of Zfp462 protein in osteoclast and osteoblast precursors (Figure S1E, F).

A previous *Zfp462^−/−^* knockout mouse model described by Wang et al. ([Wang et al., 2017](#_ENREF_14)) exhibited embryonic lethality due to neural tube defects, whereas our *Zfp462^−/−^* mice were viable and did not display embryonic lethality. These differences are likely attributable to variation in genetic background between strains ([Davidson, Li, Churchill, Osborne, & McDermid, 2007](#_ENREF_3); [Leduc, Singh, & McDermid, 2017](#_ENREF_9)) and to distinct allele-generation strategies, including potential insertional or regulatory effects of the PiggyBac-based approach ([Cadinanos & Bradley, 2007](#_ENREF_2); [Shearwin, Callen, & Egan, 2005](#_ENREF_12)).

**Generation of conditional *Zfp462^f/f^* mice**

To generate *Zfp462^f/f^* mice, two single guide RNA (sgRNAs) along with two single-stranded oligodeoxynucleotides (ssODNs) containing LoxP sequences were used to insert LoxP sites flanking exon 5 of *Zfp462* gene. Particularly, after fertilized eggs were obtained from the oviducts of 3–4-week-old female C57BL/6J mice that had undergone superovulation and mating, the cytoplasm of pronuclear-stage embryos was co-microinjected with the sgRNAs, Cas9 mRNA, and ssODNs. After 24 h of incubation at 37ºC, two-cell stage embryos were transferred to pseudopregnant ICR foster mothers. *Zfp462^f/f^* mice were mated with osteoblast-specific *Col2.3-cre* mice (Riken, RBRC05603) to generate *Col2.3;Zfp462^f/f^* mice. To assess Zfp462 knockout efficiency in osteoblasts, bone marrow cells from mouse tibias were differentiated into osteoblasts with osteogenic medium for 21 days and then subjected to qRT-PCR or western blotting.

**Genotyping**

For genotyping, genomic DNA was extracted from tail tissues and amplified using PCR with a Taq DNA polymerase (SolGent). The following primers were used: *Zfp462^−/−^* sense, 5′-GAG GGT AGC CCC CGA TCA T-3′ and antisense, 5′-TTG TCC TCG GCA ACA TCA GTT-3′; Col2.3-cre sense, 5′-CTC TGG CTG TGG CCA CAG CTG AC-3′ and antisense, 5′-TCC GGT TAT TCA ACT TGC ACC ATG C-3′; and *Zfp462^flox^* sense, 5′-AAC CAG AGT AAC CAC CAG TGC-3′ and antisense, 5′-ACC CAC CGT TTG GAA TGC TAT-3′.

**Preparation of osteoblasts**

Murine osteoblastic MC3T3-E1 cells, human osteoblastic hFOB 1.19 cells, human osteosarcoma U2OS cells, and human HEK293T cells were purchased from the American Type Culture Collection. MC3T3-E1 cells were cultured in alpha modified Eagle’s minimum essential medium (α-MEM) containing 10% fetal bovine serum (FBS). hFOB 1.19 cells were maintained in a 1:1 mixture of Ham’s F12 medium and Dulbecco’s modified Eagle’s medium (DMEM) with 10% FBS and 0.3 mg/mL G418 (Sigma-Aldrich, 4727878001) at 33.5°C for cell growth and 39.5°C for differentiation. U2OS and HEK293T cells were maintained in DMEM with 10% FBS.

For calvarial osteoblasts, calvarial bones from newborn mice were digested with α-MEM containing 0.1% collagenase (Gibco, 17018-029) and trypsin (Gibco, 25200-056). The isolated primary calvarial osteoblast precursors were differentiated into osteoblasts with 50 μg/mL ascorbic acid (Sigma-Aldrich, A4544) and 10 mM β-glycerophosphate (Sigma-Aldrich, G9422). Culture medium was replaced every 2–3 days. MC3T3-E1 and hFOB 1.19 cells were also induced to differentiate into osteoblasts using this protocol.

Human BMSCs were isolated from surgically discarded bones (ribs, femurs, and acetabula) of patients without bone disease (Table S4) as previously described ([G. S. Kim et al., 2003](#_ENREF_7)). Briefly, bones were excised and collected bone marrow was centrifuged at 1400 rpm for 10 min. Cell pellets were resuspended in α-MEM containing 10% FBS, and BMSC fraction was obtained using Ficoll/Hypaque gradient (specific gravity 1.077 g/cm^3^) centrifugation according to the manufacturer’s protocol (Nycomed Pharma).

**Osteoclast differentiation and TRAP staining**

Bone marrow cells from mouse femurs and tibias were treated with red blood cell lysis buffer (Sigma-Aldrich, R7757), centrifuged at 1500 rpm for 5 min, and incubated overnight in α-MEM containing 10% FBS. Non-adherent cells were collected and cultured in α-MEM containing 10% FBS, 30 ng/mL M-CSF (R&D Systems, 416-ML-050), and 100 ng/mL RANKL (R&D Systems, 462-TEC-010) to induce osteoclast differentiation.

TRAP staining was performed as previously described ([Takahashi, Udagawa, Kobayashi, & Suda, 2007](#_ENREF_13)). Briefly, 10 mg naphthol AS-MX phosphate (Sigma-Aldrich, N4875) was dissolved in 1 mL of N,N′-dimethylformamide (Sigma-Aldrich, D4451) to create Reagent A. Reagent B was prepared by dissolving 11.5 g sodium L-tartrate dibasic dehydrate (Sigma-Aldrich, S4797) in 1 L of 50 mM sodium acetate buffer, pH 5.0. TRAP staining solution was created by dissolving 10 mg fast red violet LB salt (Sigma-Aldrich, F3381) in a mixture of 833 μL Reagent A and 20 mL Reagent B, followed by filtration. Osteoclasts were fixed with 4% paraformaldehyde, incubated with TRAP staining solution for 15–30 min at 37°C, and subjected to light microscopy. TRAP-positive multinuclear cells (≥3 nuclei) with a complete actin ring were counted as osteoclasts.

**Immunofluorescence analysis and immunohistomorphometry of murine bones**

Murine femurs were fixed in 4% paraformaldehyde, decalcified in Osteosoft (Sigma-Aldrich, 101728), and sectioned into 5-μm-thick slices using a cryostat. The paraffin-embedded bone sections were deparaffinized, rehydrated, and subjected to antigen retrieval with a citrate buffer (10 mM citric acid, pH 6.0) at 90–95°C for 15 min. The sections were incubated overnight with an anti-osteocalcin antibody (1/200 dilution, Takara, M173) at 4°C, followed by Alexa Fluor 647-conjugated secondary antibody (1/250 dilution, ThermoFisher Scientific A-21244) for 1 h at room temperature. Nuclei were counterstained with DAPI, and fluorescence was detected using a Carl Zeiss LSM 710 confocal microscope (Carl Zeiss). For immunohistomorphometry, paraffin-embedded bone sections (5 μm thick) were incubated with TRAP staining solution and toluidine blue solution and photographed using the Olympus BX53 microscope (Olympus Corporation). Toluidine blue is a basic thiazine metachromatic dye often used to identify osteoblasts, which appear as cuboidal mononuclear cells on the bone surface. A bone surface bearing four adjacent toluidine blue-labeled cuboidal cells was defined as an osteoblast-populated surface ([Henry & Bordoni, 2024](#_ENREF_4)).

**Preparation and immunohistochemistry of human femurs**

Human proximal femur bones were collected from patients undergoing orthopedic surgery. Patients with osteoporotic hip fractures were classified as the fracture group, whereas those with degenerative arthritis served as the control group. Details regarding patient height, weight, and bone mineral density were retrieved from their medical records. The femurs were fixed in 4% paraformaldehyde solution at 4°C for 24 h, decalcified with 10% EDTA solution, dehydrated with ethanol, and embedded in paraffin. The paraffin-tissue sections were then deparaffinized in xylene, rehydrated with graded ethanol solutions (100%, 95%, and 80%), incubated with 10 mM sodium citrate buffer (pH 6.0) at 90–95°C for 15 min. After cooling for 30 min at room temperature, the sections were incubated with 1% hydrogen peroxide in methanol for 20 min at room temperature and incubated overnight with anti-ZNF462 antibody (1/50 dilution, Sigma-Aldrich, HPA022283) at 4°C. These sections were then washed, incubated with the Dako Envision + System-horseradish peroxidase-labeled polymer secondary antibody system (Dako, K4003) for 30 min at room temperature, and then stained using the Dako Liquid DAB + substrate chromogen system (Dako, K3467) according to manufacturer’s instructions. The slides were then viewed under an Olympus BX53 microscope imaging system (Olympus Corporation).

**ALP activity and *in vitro* mineralization assays**

To measure ALP activity in osteoblasts, calvarial cells (2.5 × 10^4^) were seeded into a 12-well culture plate, cultured in α-MEM for 2–3 days, and differentiated in α-MEM containing 50 μg/mL ascorbic acid and 10 mM β-glycerophosphate for 3 or 6 days. After washing with phosphate-buffered saline (PBS), the cells were lysed in 0.4 mL lysis buffer (50 mM Tris-HCl, pH 7.4), sonicated on ice (twice for 30 s) and centrifuged for 10 min at 13,000 rpm at 4°C. The supernatant was incubated with p-nitrophenylphosphate substrate (Sigma-Aldrich) for 15–30 min at 37°C, and absorbance was measured at 405 nm using a microplate reader (Tecan Group Inc.). Protein concentration in the cell lysates was determined using the BCA assay kit (Invitrogen) according to manufacturer protocols. ALP activity was expressed as absorbance at 405 nm (OD value)/min/mg of protein.

To measure mineralization in osteoblasts, cells were washed with PBS, fixed in ice-cold 70% ethanol for 30 min at 4°C, again washed with PBS, and stained with 40 mM alizarin-red S solution (pH 4.2) for 10 min at room temperature. After rinsing twice with deionized water, alizarin-red S staining was solubilized in 10% cetylpyridinium chloride (Sigma-Aldrich), and absorbance was detected at 570 nm with the microplate reader (Tecan Group Inc.).

**Cell counts**

Cells (4 × 10^4^) were seeded into a 12-well culture plate, cultured in α-MEM for 3 or 6 days, trypsinized, resuspended in α-MEM, stained with 0.5% trypan blue (Sigma-Aldrich), and counted using a hemocytometer (Marienfeld Superior).

***In vitro* bone-resorption assay**

To conduct bone resorption assay, bone marrow cells were seeded onto dentin slices (IDS Ltd.) and cultured in α-MEM containing 10% FBS, 30 ng/mL M-CSF, and 100 ng/mL RANKL for 10 days. The cells were then removed by rubbing the slices. The slices were stained with hematoxylin (Sigma-Aldrich) and photographed using the Olympus BX53 microscope (Olympus Corporation). Resorbed bone area was quantified using Image Pro plus program (version 6.0; Media Cybernetics Inc.).

**Micro-CT analysis**

Femur bones were excised from euthanized mice, fixed with 4% paraformaldehyde for 24 h at 4°C, and subjected to micro-CT scanning using a Skyscan 1172 scanner (Bruker) at 70 kV and 126 μA with a 6.48-μm pixel size. Trabecular bone in the distal femoral metaphysis and cortical bone at the femoral midshaft were analyzed by establishing regions of interest that extended 3 mm from the femoral growth plate to the proximal metaphysis. Three-dimensional algorithms in CT analyzer software (version 1.16.1.0; CTAn, Skyscan) were used to measure relevant bone morphometric variables.

**Bone formation rate**

WT and *Zfp462^−/−^* mice were intraperitoneally injected with 30 mg/kg calcein and 30 mg/kg alizarin red S at 3 or 5 days before sacrifice, respectively. Femurs were cut into 20-μm-thick sections. Images were captured using a fluorescence microscope (Carl Zeiss) and analyzed using a semiautomatic image analysis system (Histometry RT Digitizer) and Bio-Quant program (version 19.9.60; Bio-Quant, Inc.).

**ELISA of serum bone turnover markers**

Serum was harvested from each mouse just before sacrifice. Two bone formation markers were measured: P1NP concentrations were determined using a P1NP ELISA kit (IDS, AC-33F1) and ALP activity was assessed using a mouse bone ALP activity ELISA kit (Mybiosource, MBS163883). One bone resorption marker was quantified in serum CTX levels using a CTX ELISA kit (IDS, AC-06F1).

**RNA interference**

DNA oligonucleotides encoding sh-RNAs specific to human ZNF462, RUNX2, MOZ, and their mouse homologs (Table S5) were annealed and ligated into the lentiviral vector pLKO.1 (Addgene) according to reported procedures (https://www.addgene.org/tools/protocols/plko/). To collect lentiviral particles, HEK293T cells were transfected with constructs encoding VSV-G (envelope plasmid), NL-BH (packaging plasmid), and each sh-RNA using Lipofectamine 3000 reagent (Invitrogen) as per manufacturer’s protocol. MC3T3-E1 and hFOB 1.19 cells were infected with each sh-RNA lentivirus for 24 h and then selected with puromycin (2 μg/mL) for 2 weeks. Primary calvarial osteoblast precursors were transiently transfected with each sh-RNA.

**qRT-PCR**

RNA was prepared from calvarial osteoblasts using TRIzol according to the manufacturer’s instructions. The isolated RNA (1 μg) was transcribed into cDNA using the Superscript First-strand synthesis system (Invitrogen). Real-time PCR was conducted using the Light cycler SYBR Green RT-PCR kit (Roche, 04887352001). The primers used for real-time PCR assay are described in Table S6. For TaqMan assay, Zfp462 probes were labeled with the reporter dye 6-carboxyfluorescein (FAM) and quencher dye 6-carboxytetramethylrodamine (TAMRA) at its 5′ and 3′ end, respectively. Real-time PCR was performed using TaqMan gene expression master mix (ThermoFisher Scientific, 4369016) according to the manufacturer’s protocols.

**ChIP assay**

ChIP assays were performed according to established protocols ([J. M. Kim et al., 2015](#_ENREF_8)). Briefly, cells cultured in a 100-mm culture dish were cross-linked by adding 270 μL of 37% formaldehyde for 10 min at room temperature, followed by adding 1 mL of 1.25 M glycine for 5 min. Cells were washed with PBS and lysed in 1 mL hypotonic buffer (10 mM Hepes-KOH, pH 7.8, 10 mM KCl, and 1.5 mM MgCl_2_) containing 1× protease inhibitor on ice for 10 min with occasional mixing by gently tapping the tube. NP-40 was added to a final concentration of 0.3%. Cell lysates were centrifuged at 13,000 rpm for 1 min to obtain nuclear pellets. The pellets were resuspended in 0.4 mL nucleus lysis buffer (50 mM Tris-HCl, pH 8.0, 10 mM EDTA, and 1% SDS) containing 1× protease inhibitor and incubated on ice for 60 min with occasional mixing. Nuclear cell fractions were sonicated to shear the chromatin. After centrifugation at 13,000 rpm for 5 min, 100 μL of the supernatant was diluted with 900 μL dilution buffer (20 mM Tris-HCl, pH 8.0, 167 mM NaCl, 1.2 mM EDTA, 0.01% SDS, and 1% Triton X-100). The diluted samples were incubated with 5–10 μg of anti-Runx2, anti-Zfp462, anti-MOZ, anti-ZNF462, anti-H3, anti-H3K9Ac, anti-H3K14Ac, anti-H3K23Ac, anti-H2A.Z, anti-mH2A1, anti-H3K4me3, anti-H3K27me3, and anti-normal rabbit IgG antibodies at 4°C overnight, after which the mixtures were incubated with protein A/G agarose beads (Santa Cruz Biotechnology sc2003) at 4°C for 4 h. The immunoprecipitated chromatin was then decrosslinked at 65°C overnight and the purified DNA was analyzed by real-time PCR using primers that amplify the promoter or coding regions of *Alpl*/*ALPL*, *Sema7a*/*SEMA7A*, *Spp1*/*SPP1*, and *Zfp462*/*ZNF462* genes. ChIP–qPCR quantification was performed using the percent (%) input method. Purified input DNA was serially diluted to generate a standard curve, and qPCR was performed on both input standards and ChIP DNA samples. The percent input for each ChIP sample was calculated from the standard curve based on Ct values, and all values were normalized to input. The primers used for quantitative PCR are listed in Table S7.

**Reporter assay**

Cells were seeded into 24-well culture plates for 24 h and transfected with 100 ng of 6 concatenated osteoblast-specific elements cassettes (6×OSE-luc) and various expression constructs using Lipofectamine 3000 (ThermoFisher Scientific). After 48 h, the cells were collected and subjected to Dual-Luciferase reporter assay system (Promega, E1910) according to the manufacturer’s instructions.

**Immunoprecipitation**

Cells were lysed in a lysis buffer (50 mM Tris-HCl, pH7.3, 150 mM NaCl, 1 mM EDTA, 0.1% NP-40, and 1 mM PMSF) containing 1× protease inhibitor. Total cell extracts were incubated with anti-ZNF462, anti-Zfp462 antibodies, or control IgG at 4°C overnight, followed by incubation with protein A/G agarose beads (Santacruz biotechnology). The beads were washed with a lysis buffer and bound ZNF462 and Zfp462 were analyzed using western blot with the indicated antibodies. To assess exogenous protein interactions, HEK293T cells were seeded into 100-mm culture dish and cultured for 24 h. The cells were transfected with FLAG-ZNF462, HA-Runx2, or His-MOZ constructs using Lipofectamine 3000 (ThermoFisher Scientific). After 48 h, the cells were lysed with a lysis buffer and subjected to immunoprecipitation with anti-FLAG or anti-HA antibodies at 4°C overnight followed by incubation with protein A/G agarose beads. The beads were washed four times with lysis buffer and resuspended in SDS sample buffer for western blot analysis.

**Histone extraction and H3/H4 acetylation**

Histone H3 and H4 acetylation was measured using the Epiquik global histone H3 (Epigentek, P4008) and H4 (Epigentek, P4009) acetylation assay kits. Briefly, cells were harvested and resuspended in diluted lysis buffer on ice for 5 min with vortexing. After centrifugation, lysis buffer was added to the samples, followed by three volumes of extraction buffer with glycerol solution on ice for 5 min with vortexing. After another centrifugation, trichloroacetic acid was added to the supernatant to a final concentration of 25%, followed by incubation on ice for 30 min. The precipitate was collected by centrifuging the solution at 12,000 rpm for 10 min at 4°C and then washed with acetone containing 0.1% HCl. After a final centrifugation, the resulting pellet (histone extract) was dissolved in distilled water, and histone buffer was added to achieve a final concentration of 400 ng/μL. The histone extract (2 μg) was added to each well of the assay strip and incubated at 37°C for 60–90 min to allow the solution to evaporate. Blocking buffer (150 μL) was then added to each dried well and incubated at 37°C for 30 min. After washing, 50 μL capture antibody (1 μg/mL) was added to each well, followed by incubation for 1 h at room temperature. After further washing, each well was incubated with 50 μL detection antibody (0.4 μg/mL) for 30 min at room temperature. Each well was then treated with 100 μL developing solution for 2–10 min at room temperature, after which 50 μL stop solution was added. Absorbance at 450 nm was measured using the microplate reader (Tecan Group Inc.).

**Runx2 DNA-binding activity**

Runx2 DNA-binding activity was determined using the Runx2 transcription factor assay kit (Abcam, ab207193). Briefly, cells were collected and resuspended in a hypotonic buffer (20 mM Hepes, pH 7.5, 5 mM NaF, 10 μM Na_2_MoO_4_, and 0.1 mM EDTA) on ice for 15 min. NP-40 (10%) was then added to achieve a final concentration of 0.5% and mixed via pipetting. The nuclear pellet was obtained, resuspended in 50 μL complete lysis buffer on ice for 30 min with gentle rocking, and centrifuged at 14,000 ×*g* at 4°C. The supernatant containing the nuclear extract was harvested. Thereafter, 30 μL complete-binding buffer containing WT or mutated oligonucleotides (20 pmol/well) was added to each well in an assay strip, after which 20 μL nuclear extract (20 μg/well) was added. The wells were incubated for 1 h at room temperature and washed three times with wash buffer, followed by the addition of 100 μL anti-Runx2 antibody (1/1000 dilution). After incubating for 1 h at room temperature, the wells were washed and 100 μL anti-rabbit HRP antibody (1/1000 dilution) was added. Following 1-h incubation at room temperature, the wells were washed four times with wash buffer and incubated with 100 μL developing solution for 2–10 min. Finally, after adding 100 μL stop solution per well, absorbance at 450 nm was read using the microplate reader (Tecan Group Inc.).

**RNA sequencing**

Total RNA was isolated from differentiated osteoblasts using the TRIzol reagent (Invitrogen) according to the manufacturer’s instructions. Total RNA concentration was determined using Quant-IT RiboGreen (Invitrogen, #R11490). To evaluate the integrity of total RNA, samples were analyzed using the TapeStation RNA screentape (Agilent). Only high-quality RNA samples with an RNA integrity number greater than 7.0 were used to construct RNA libraries. Libraries were constructed using total RNA (1 μg) and Illumina TruSeq mRNA Sample Prep kit (Illumina Inc.). Cleaved RNA fragments were reverse transcribed into first-strand cDNA using SuperScript II reverse transcriptase (Invitrogen) and random primers, followed by second-strand cDNA synthesis using DNA Polymerase I and RNase H. Library quantification was conducted using the KAPA Library Quantification protocol guide, and quality was assessed using the TapeStation D1000 ScreenTape (Agilent Technologies). Indexed DNA libraries were sequenced with 2× 100-bp paired-end sequencing on an Illumina HiSeq4000 (Illumina, Inc.) according to the manufacturer’s protocol. Differentially expressed genes (*P*-value < 0.05; fold change >2) were functionally analyzed in the context of gene ontology using the DAVID online tool (https://david-d.ncifcrf.gov/). GSEA was performed using GSEA platform (http://www.broad.mit.edu/gsea; Broad Institute).

**Constructs and protein-protein binding assay**

FLAG-tagged ZNF462 (RC224316) and His-tagged MOZ (0Hu24263C) plasmids were purchased from OriGene Technologies Inc. and GenScript, respectively. Mutant HA-tagged MOZ and HA-tagged ZNF462 expression vectors were generated by amplifying the corresponding cDNAs (Nhe I-MOZ mutants-Not I and Not I-ZNF462 mutants-Mfe I) via PCR and inserting them into the correct reading frames of the mammalian expression vector pIRES containing HA coding sequences. To generate mutant histone H3 mammalian expression vectors, histone H3 cDNAs were mutated using the Quickchange II site-directed mutagenesis kit (Agilent Technologies). For *in vitro* binding assays, HEK293T cells were transfected with full-length WT plasmids (FLAG-ZNF462, His-MOZ, and FLAG-Runx2) and HA-tagged MOZ/ZNF462 deletion mutant plasmids. After 48 h, total cell extracts were obtained using lysis buffer (50 mM Tris-HCl, pH 8.0, 150 mM NaCl, 1 mM EDTA, 0.5% sodium deoxycholate, 0.1% SDS, and 1% NP-40). The cell lysates were treated with an anti-HA antibody (Cell Signaling, 2362) and then incubated with protein A/G agarose beads with gentle rotation at 4°C overnight. After removing the supernatant, the sample pellets were separated on SDS-PAGE and analyzed via western blot using anti-FLAG (Sigma-Aldrich, F1804), anti-HA (Cell signaling, 2362), and anti-His (Cell signaling, 12698) antibodies.

**Three-point bending analysis**

Murine tibias from 16-week-old female mice were fixed in 4% paraformaldehyde for 24 h and stored at −20 °C in 70% ethanol. After washing with PBS, each tibia was placed perpendicularly on the angled top of a triangular supporting pin so that the tibia remained suspended. Three-point bending analysis was not feasible at 4 weeks of age, consistent with previous reports ([Jepsen, Silva, Vashishth, Guo, & van der Meulen, 2015](#_ENREF_5); [Schriefer et al., 2005](#_ENREF_11)). A universal testing machine (ST-1002, SALT) was used to apply force to the mid-shaft of the tibia. A loading bar was moved downward at a crosshead speed of 0.167 mm/s onto the middle of the tibia until fracture. The force–deformation curve was used to calculate the ultimate force (F) required to break the bone (Newtons, N). Maximum load (N) and stiffness (N/mm) were determined from the load–displacement diagram using the universal testing machine software (version 12.6.0; SALT).

***Ex vivo* calvaria culture**

Calvariae isolated from 4-day-old mice were cultured in a 12-well culture plate with α-MEM for 24 h. The samples were then treated with control or *Moz* sh-RNAs for an additional 24 h and incubated in BGJb medium (Gibco, 12591038) containing 0.1% bovine serum albumin, 50 μg/mL ascorbic acid, and 10 mM β-glycerophosphate for 9 days as previously described ([Mohammad, Chirgwin, & Guise, 2008](#_ENREF_10)). The culture medium was changed every other day. The calvariae were then fixed in 4% paraformaldehyde for 24 h, decalcified in 10% EDTA for 24 h at 4°C, embedded in paraffin, and sectioned into 5-μm-thick slices. The bone sections were deparaffinized, rehydrated, and stained with hematoxylin and eosin. Images of the calvarial sections were acquired using a digital slide scanner system (VS200, Olympus) and analyzed using Olympus OlyVIA software (version 3.3; Olympus) and Image Pro Plus (version 6.0; Media Cybernetics).

**Western blot analysis and antibodies**

Cells were lysed in RIPA cell lysis buffer (Enzo Life Sciences, ADI-80-1284) containing 1× protease inhibitor (Sigma-Aldrich, P8340). The samples were separated by sodium dodecyl sulfate (SDS)-polyacrylamide gel electrophoresis (PAGE) and transferred onto nitrocellulose membranes. The membranes were blocked with 5% skim milk for 1 h and then incubated with primary antibody at 4°C overnight. After washing with phosphate-buffered saline containing 0.05% Tween 20, membranes were incubated with horse radish peroxidase (HRP)-conjugated secondary antibody for 1 h at room temperature, then developed with enhanced chemiluminescence reagents (Dynebio ECL Dura, GBE-D50 or Pico plus, GBE-P100) and detected on a film. Band density was quantified using an Image Gauge program (version 4.0; Fujifilm). The antibodies used are as follows: human ZNF462 (A303-270A) and Mybbp1a (A301-327A) antibodies from Bethyl Laboratories; Runx2 (sc-390351) and Rankl (sc-7627) antibodies from Santa Cruz Biotechnology; Actin (A3854), H3K27me3 (07-449), G9A (G6919), ZNF462 (HPA022283), and FLAG (F1804) antibodies from Sigma-Aldrich; Runx2 (12556), HA (2362), H3 (9715 for western blot and 4620 for ChIP), H3K9Ac (9648), H3K14Ac (7627), FLAG (14793), and His (12698) antibodies from Cell signaling; MOZ (61327 for ChIP), H2A.Z (39113), and PARP1 (39561) antibodies from Active Motif; Atp6v0d2 (ab321809), H3K23Ac (ab177275), anti-H3K4me3 (ab12209), mH2A1 (ab37264), mH2A2 (ab102126), and Pol II (ab817) antibodies from Abcam; MOZ (AP52734PU-N for western blot) antibody from OriGene; and osteocalcin (M173) antibody from Takara. A mouse Zfp462 polyclonal antibody was developed by immunizing rabbits with a synthetic peptide from Zfp462 (amino acids 2447–2474: EESMQLPSIEAKEDDEPIGIDFPLKSET) (GW Vitek Company).

**LC-MS analysis and database search**

The peptide mixture in each sample was reconstituted in 0.1% formic acid and peptide separation was performed using the Ultimate3000 RSLC system coupled with a Q Exactive BioPharm version mass spectrometer (ThermoFisher Scientific). The liquid-chromatography gradient and data-dependent acquisition-MS were performed according to previously published methods ([Ahn et al., 2020](#_ENREF_1); [E. Y. Kim et al., 2020](#_ENREF_6)). The resulting MS spectra were searched using Sequest HT on Proteome discoverer (version 2.3; ThermoFisher Scientific) against the SwissProt human proteome sequence database (May 2018). Label-free quantities of each protein were determined and used for further statistical analysis.

**Identification of ZNF462-interacting factors**

U2OS cells were transfected with FLAG-tagged ZNF462 plasmid using Lipofectamine 3000. After 48 h, ectopic FLAG-ZNF462 and its interacting proteins were purified by immunoprecipitation using anti-FLAG antibody in a binding buffer (20 mM Tris-HCl, pH 7.3, 300 mM KCl, 0.2 mM EDTA, 20% glycerol, and 0.1% NP-40). The purified proteins were resolved by 4%–20% gradient SDS-PAGE and stained with silver staining. Major protein bands were excised, washed with 500 mL distilled water, and destained with 50 mM sodium thiosulfate and 15 mM potassium ferricyanide. The gel was then dehydrated in 100% acetonitrile and rehydrated with 100 mM ammonium bicarbonate. This dehydration and rehydration process was repeated three times and then followed by a single dehydration in 100% acetonitrile. Subsequently, the gel pieces were dried and rehydrated at 47℃ for 45 min in digestion buffer containing mass grade trypsin/LysC in 50 mM ammonium bicarbonate at a concentration of 0.01 mg/mL (Promega) and incubated overnight at 37℃. The digested peptide solutions were moved to new tubes, dried, and kept at −80℃ until LC-MS analysis.

**Tissue microarrays**

Bone marrow tissue microarrays (BM481), which consisted of two samples each from 6 young (<50 years) and 18 older (≥50 years) patients (Table S3), were obtained from U.S. Biomax Inc. The paraffin-embedded sections were deparaffinized, rehydrated, subjected to antigen retrieval with 10 mM citrate buffer (pH 6.0), incubated with 3% hydrogen peroxide solution for 30 min at room temperature, and then blocked with 10% normal goat serum (Abcam, ab7481) for 1 h at room temperature. Thereafter, the sections were incubated overnight with anti-ZNF462 (1/50 dilution, Bethyl Laboratories, A303-270A), anti-H2A.Z (1/50 dilution, Active motif, 39113), and anti-mH2A1 (1/50 dilution, Abcam, ab37264) antibodies at 4°C. After washing, the section was incubated with peroxidase-labeled polymer conjugated to goat anti-rabbit IgG (Dako, K4003) for 30 min at room temperature. The signal was developed using Envision DAB + Substrate Chromogen system (Dako, K3467) and counterstained with hematoxylin. The stained sections were mounted, and images were captured using the Olympus BX53 microscope (Olympus Corporation). The relative staining indexes were calculated using ImageJ program (version 1.54g; https://imagej.net/ij/).

**DNA methylation analysis**

Genomic DNA from osteoblasts and osteoclasts was isolated using DNeasy blood & tissue kit (Qiagen, 69504). The harvested DNA (2 μg) was modified by bisulfite treatment using the EZ DNA methylation kit (Zymo Research, D5001) according to the manufacturer’s instructions. The modified genomic DNA was then amplified by PCR using the following specific primers: sense, 5′-GAG AGA GAY GGA TAT TTT AGG TTA TTT GTA GTT GTA-3′ and antisense, 5′-CTC CTC CCC RAA ACA CTC AAA CCA CCC-3′. The PCR products were cloned into the pGEM-T easy vector (Promega, A1360), and five clones from each sample were sequenced using T7 and SP6 universal primers. QUMA online tool (<http://quma.cdb.riken.jp/>) was used to analyze DNA methylation.

**Blood Tests**

Blood was collected from 16-week-old male WT and *Zfp462^−/−^* mice and centrifuged to obtain plasma and serum. Hematological variables in plasma were measured using the ADVIA 2120i Hematology System (Siemens Healthcare Diagnostics). Biochemical variables in serum were analyzed using the Hitachi 7180 Automatic Analyzer (Hitachi High Technologies).

**Table** **S1**. Clinical characteristics of patients with and without osteoporotic hip fractures.

|  | Without fractures (*n* = 5) | With fracture (*n* = 4) | *p*-value |
| --- | --- | --- | --- |
| Sex (male/female) | 1/4 | 1/3 |  |
| Age (y) | 60.0 ± 7.3 | 80.8 ± 9.3 | 0.059 |
| Height (cm) | 158.5 ± 5.1 | 158.8 ± 6.5 | 0.467 |
| Weight (kg) | 62.6 ± 6.5 | 53.2 ± 8.4 | 0.200 |
| Body mass index (kg/m^2^) | 24.7 ± 1.8 | 20.7 ± 1.8 | 0.084 |
| Bone mineral density at lumbar spine (g/cm^2^) | 1.008 ± 0.068 | 0.683 ± 0.055 | 0.009 |
| Bone mineral density at femur neck (g/cm^2^) | 0.789 ± 0.070 | 0.612 ± 0.096 | 0.084 |
| Bone mineral density at total femur (g/cm^2^) | 0.989 ± 0.078 | 0.746 ± 0.151 | 0.095 |

**Table** **S2**. Blood tests in male wild-type (WT) and *Zfp462^−/−^* mice at 16 weeks of age.

|  | WT (*n* = 4) | *Zfp462^−/−^* (*n* = 6) |
| --- | --- | --- |
| White blood cells (× 10^3^/μL) | 6.73 ± 1.13 | 7.60 ± 2.25 |
| Red blood cells (× 10^6^/μL) | 9.23 ± 0.80 | 9.60 ± 0.50 |
| Hemoglobin (g/dL) | 14.0 ± 0.2 | 14.3 ± 0.5 |
| Hematocrit (%) | 43.5 ± 2.5 | 44.9 ± 1.3 |
| Platelet (× 10^3^/μL) | 672.3 ± 388.3 | 775.0 ± 354.9 |
| Alanine aminotransferase (U/L) | 22.6 ± 2.8 | 26.5 ± 3.5 |
| Creatinine (mg/dL) | 0.46 ± 0.04 | 0.51 ± 0.05 |
| Calcium (mg/dL) | 8.71 ± 0.22 | 8.93 ± 0.3 |
| Phosphate (mg/dL) | 5.49 ± 0.87 | 5.94 ± 0.74 |

**Table** **S3**. Demographic information of subjects who provided bone marrow for human tissue arrays that were subjected to immunohistochemistry.

| Patient No. | Age (y) | Sex | Site |
| --- | --- | --- | --- |
| Patient 1 | 66 | F | Bone marrow |
| Patient 2 | 62 | M | Bone marrow |
| Patient 3 | 66 | M | Bone marrow |
| Patient 4 | 53 | M | Bone marrow |
| Patient 5 | 62 | M | Bone marrow |
| Patient 6 | 36 | M | Bone marrow |
| Patient 7 | 30 | M | Bone marrow |
| Patient 8 | 61 | F | Bone marrow |
| Patient 9 | 72 | M | Bone marrow |
| Patient 10 | 62 | F | Bone marrow |
| Patient 11 | 71 | M | Bone marrow |
| Patient 12 | 45 | M | Bone marrow |
| Patient 13 | 63 | M | Bone marrow |
| Patient 14 | 43 | F | Bone marrow |
| Patient 15 | 58 | M | Bone marrow |
| Patient 16 | 75 | M | Bone marrow |
| Patient 17 | 21 | F | Bone marrow |
| Patient 18 | 66 | M | Bone marrow |
| Patient 19 | 53 | M | Bone marrow |
| Patient 20 | 41 | M | Bone marrow |
| Patient 21 | 60 | M | Bone marrow |
| Patient 22 | 75 | M | Bone marrow |
| Patient 23 | 63 | M | Bone marrow |
| Patient 24 | 50 | F | Bone marrow |

**Table** **S4**. Clinical information of subjects who provided bone marrow stromal cells.

| Patient No. | Age (y) | Sex | Site |
| --- | --- | --- | --- |
| P41 | 41 | M | Femur |
| P43 | 43 | M | Rib |
| P46 | 46 | M | Rib |
| P47 | 47 | M | Femur |
| P56 | 56 | M | Rib |
| P59 | 59 | F | Rib |
| P65 | 65 | M | Rib |
| P67 | 67 | F | Acetabulum |
| P68 | 68 | M | Acetabulum |
| P69-1 | 69 | F | Rib |
| P69-2 | 69 | M | Femur |
| P73 | 73 | M | Acetabulum |
| P74 | 74 | F | Acetabulum |

**Table** **S5**. Sequences of sh-RNAs used in the study.

| Genes | Organism | Sequences (5′- 3′) |
| --- | --- | --- |
| *ZNF462* | Human | TTAGGACTGAACAATCTATTT |
| *RUNX2* | Human | CAGCACTCCATATCTCTACTA |
| *MOZ* | Human | CGGCGCTATACTAATCCAATA |
| *Zfp462* | Mouse | CGCAACATGATCGACCACATA |
| *Runx2* | Mouse | GCACGCTATTAAATCCAAATT |
| *Moz* | Mouse | GCGTCAAAGACGGGACGATTT |

**Table S6.** Specific primer sequences for qRT-PCR.

| Primers | Organism | Forward (5′-3′) | Reverse (5′-3′) |
| --- | --- | --- | --- |
| *GPNMB* | Human | GATGTTGAAGGCGAAGCTGT | TGGCAGTTTTCATCAGGAATC |
| *Adar* | Human | ttcgagaatcccaaacaagg | ctggattccacagggattgt |
| *Ifitm1* | Human | caaagccagaagatgcacaa | gaacagggaccagacgacat |
| *Nppc* | Human | cggcctgggatgttagtg | aaagatgacctcagcacaacg |
| *BMP4* | Human | aagcgtagccctaagcatca | tggttgagttgaggtggtca |
| *Sema7a* | Human | cctttcatgtgctttacctaactaca | gatgttgaaggcgaagctgt |
| *MMP13* | Human | ccagtctccgaggagaaaca | aaaaacagctccgcatcaac |
| *Spp1* | Human | gagggcttggttgtcagc | caattctcatggtagtgagttttcc |
| *Sp7* | Human | catctgcctggctccttg | caggggactggagccata |
| *Alpl* | Human | cctgccttactaactccttagtgc | cgttggtgttgagcttctga |
| *Igf1* | Human | tgtggagacaggggctttta | atccacgatgcctgtctga |
| *ZNF462* | Human | ccgtgaatgccagtaatcag | cggtccccaatgaagttg |
| *MOZ* | Human | ggcagtgcatcgagtgtaaa | ctcggtcacatgaatcacaaa |
| *18S* | Human | GGCCCTGTAATTGGAATGAGTC | CCAAGATCCAACTACGAGCTT |
| *Gpnmb* | Mouse | ACGGCAGGTGGAAGGACT | CGGTGAGTCACTGGTCAGG |
| *Adar* | Mouse | tctttgaaaatcccaagcaag | cactggactccacaggaattg |
| *Ifitm1* | Mouse | gctcctcgaccacacctct | gaccccagtacaaccacctc |
| *Nppc* | Mouse | gagcggtctgggatgttagt | tcagtgcacagagcagttcc |
| *Bmp4* | Mouse | gaggagtttccatcacgaaga | gctctgccgaggagatca |
| *Sema7a* | Mouse | ggagagaccttccatgtgct | aagacaaagctatggtcctggt |
| *Mmp13* | Mouse | TGGACCTTCTGGTCTTCTGG | GGCATCCCCACCATAGTTT |
| *Spp1* | Mouse | TCGATAGTCAAGCAAGTTCCAA | TGTCCTTGTGGCTGTGAAAC |
| *Sp7* | Mouse | CTCCTGCAGGCAGTCCTC | GGGAAGGGTGGGTAGTCATT |
| *Alpl* | Mouse | CACGGCCATCCTATATGGTAA | GAGACATTTTCCCGTTCACC |
| *Igf1* | Mouse | TGGAGTGGGAAAAGCAAGAG | CGAGAGGTGGAGTGATTTGAC |
| *Runx2* | Mouse | CCACCACTCACTACCACACG | ACTCTGGCTTTGGGAAGAGC |
| *Trap* | Mouse | CTGGAGTGCACGATGCCAGCGACA | TCCGTGCTCGGCGATGGACCAGA |
| *Nfatc1* | Mouse | GAGACAGACATCGGGAGGAAGA | GTGGGATGTGAACTCGGAAGA |
| *Atp6v0d2* | Mouse | TCAGATCTCTTCAAGGCTGTGCTG | GTGCCAAATGAGTTCAGAGTGATG |
| *Rankl* | Mouse | AGCGCAGATGGATCCTAACA | GAGTCCTGCAAATCTGCGTT |
| *Osteocalcin* | Mouse | GCTACCTTGGAGCCTCAGTC | CTCGTCACAAGCAGGGTTAAG |
| *H2A.X* | Mouse | ccgtttctaattcaacaaacttca | ccaaaggctgtgtaggatgg |
| *H2A.Z* | Mouse | ttccagtggactgtatctctgtg | cgaatgcagaaatttggttg |
| *H3.3* | Mouse | aggattactggtcctcacttgg | tgcaagctctgggtttgac |
| *H2ABbd* | Mouse | gacattgggcgttggactt | tgcgatgtcacaggatgact |
| *CENPA* | Mouse | caaggaggagaccctccag | ttctgtcttctgcgcagtgt |
| *mH2A1* | Mouse | aggatccaagggaaaattgg | aggacagtgaagccgtctgt |
| *mH2A2* | Mouse | ggaccaaaggcaagtcagag | tccgaggtggaatttgatgt |
| *Zfp462* | Mouse | cggaaacatgtccagtatggt | ttgtcctctctggtaaacatgg |
| *Moz* | Mouse | taccaacgtaagggctacgg | gtccagcattcgtaggtggt |
| *Setd1a* | Mouse | TCAAGAGTTGGCCTGAGGAG | GTGGCCTCCTCTTCTTCAGT |
| *Setd1b* | Mouse | CCGTTCTCCTATCCATCCCC | GGCTCAGGGAAAGTAGGTGT |
| *Kmt2a* | Mouse | CCATGAGATTCCGGCACTTG | AAAAGACCCCGACCATGGAT |
| *Kmt2b* | Mouse | AACATTTGCTTGGCTCCCTG | TGATCTTGGATGTGGCAGGT |
| *Kmt2c* | Mouse | CCCTTTCCAGAGAGCCAGAA | TTCTGTTTCCGTCGTCTCCA |
| *Kmt2d* | Mouse | AGAGGTGTTGAGTGGGGATG | ATGTCCTCCAGCTTGCTCTT |
| *18S* | Mouse | CTCAACACGGGAAACCTCAC | CGCTCCACCAACTAAGAACG |

**Table S7.** Specific primer sequences for for ChIP-qPCR.

| Primers | Organism | Forward (5′-3′) | Reverse (5′-3′) |
| --- | --- | --- | --- |
| *ALPL*  −1935 to −1825 | Human | gtggtggtgcacacctgtag | gctggaatgcagtagcatga |
| *ALPL*  −1181 to −1000 | Human | tgagactggcatggagtgag | gaattctctgggcaacttcg |
| *SEMA7A*  −2254 to −2146 | Human | tcccctgaatgtggaatcat | agtgatgggaagtggaccag |
| *SEMA7A*  −1504 to −1281 | Human | cccttgtctgtgccttgatt | ctgaggtacagggagggaca |
| *SPP1*  −784 to −661 | Human | cgcagagcatttgcatctaa | aaatcgcccagcctacctat |
| *SPP1*  −214 to −56 | Human | ttcatgggatccctaagtgc | acttccccctctggttttgt |
| *Alpl*  −2967 to −2781 | Mouse | aggctggcttcaaactcaaa | cacttcctttgtgcctgtca |
| *Alpl*  −1589 to −1351 | Mouse | ggctccgtggtaagagacag | ggcagtcctcgtctgtaagg |
| *Sema7a*  −1758 to −1593 | Mouse | aatgatgtaggcggaagtgg | ggcagttatgccctccagta |
| *Sema7a*  −616 to −335 | Mouse | ggaagggaggagaaaggaga | cagcgcatggatgagtctaa |
| *Spp1*  −1502 to −1348 | Mouse | taaaagcagggtttggcaag | tgccctgcagttacttcctt |
| *Spp1*  −762 to −534 | Mouse | cagagcaacaaggttcacga | ctagcctcggctccatactg |
| *ZNF462*, A | Human | ctggaagtgggtgaaatcgt | tgggcaagtctgtcctcttt |
| *ZNF462*, B | Human | ggcctctagatggatgcaag | atagcgctcccaacgtca |
| *ZNF462*, C | Human | CGGGACGCTTCTTCTgtaag | cgcttcttctcctcttccaa |
| *ZNF462*, D | Human | gtgaacagagaggcgaggag | tggcttctcctctccagaaa |
| *ZNF462*, E | Human | ccaccagaaaatggaggaga | agtgaggaaccaccacaagg |
| *Zfp462*, A | Mouse | tcaatgtgagtgggaggaga | accctgacgcacgatctaac |
| *Zfp462*, B | Mouse | aaagattggcctctagatggatac | tctaaatgctcgcaccatca |
| *Zfp462*, C | Mouse | aacgcgctcattcattcg | gcaggggacttcctctcct |
| *Zfp462*, D | Mouse | cggttgcaggaggacaac | cctcagaggagcgtgtgtaa |
| *Zfp462*, E | Mouse | agaagcatgcctgcctattc | gtgaggaaccaccacaaggt |

**Supplementary References**

Ahn, H. S., Kim, J. H., Jeong, H., Yu, J., Yeom, J., Song, S. H., . . . Kim, K. (2020). Differential Urinary Proteome Analysis for Predicting Prognosis in Type 2 Diabetes Patients with and without Renal Dysfunction. *Int J Mol Sci, 21*(12). doi:10.3390/ijms21124236

Cadinanos, J., & Bradley, A. (2007). Generation of an inducible and optimized piggyBac transposon system. *Nucleic Acids Res, 35*(12), e87. doi:10.1093/nar/gkm446

Davidson, C. E., Li, Q., Churchill, G. A., Osborne, L. R., & McDermid, H. E. (2007). Modifier locus for exencephaly in Cecr2 mutant mice is syntenic to the 10q25.3 region associated with neural tube defects in humans. *Physiol Genomics, 31*(2), 244-251. doi:10.1152/physiolgenomics.00062.2007

Henry, J. P., & Bordoni, B. (2024). Histology, Osteoblasts. In *StatPearls*. Treasure Island (FL) ineligible companies. Disclosure: Bruno Bordoni declares no relevant financial relationships with ineligible companies.

Jepsen, K. J., Silva, M. J., Vashishth, D., Guo, X. E., & van der Meulen, M. C. (2015). Establishing biomechanical mechanisms in mouse models: practical guidelines for systematically evaluating phenotypic changes in the diaphyses of long bones. *J Bone Miner Res, 30*(6), 951-966. doi:10.1002/jbmr.2539

Kim, E. Y., Ahn, H. S., Lee, M. Y., Yu, J., Yeom, J., Jeong, H., . . . Ahn, Y. M. (2020). An Exploratory Pilot Study with Plasma Protein Signatures Associated with Response of Patients with Depression to Antidepressant Treatment for 10 Weeks. *Biomedicines, 8*(11). doi:10.3390/biomedicines8110455

Kim, G. S., Hong, J. S., Kim, S. W., Koh, J. M., An, C. S., Choi, J. Y., & Cheng, S. L. (2003). Leptin induces apoptosis via ERK/cPLA2/cytochrome c pathway in human bone marrow stromal cells. *J Biol Chem, 278*(24), 21920-21929. doi:10.1074/jbc.M204598200

Kim, J. M., Kim, K., Schmidt, T., Punj, V., Tucker, H., Rice, J. C., . . . An, W. (2015). Cooperation between SMYD3 and PC4 drives a distinct transcriptional program in cancer cells. *Nucleic Acids Res, 43*(18), 8868-8883. doi:10.1093/nar/gkv874

Leduc, R. Y., Singh, P., & McDermid, H. E. (2017). Genetic backgrounds and modifier genes of NTD mouse models: An opportunity for greater understanding of the multifactorial etiology of neural tube defects. *Birth Defects Res, 109*(2), 140-152. doi:10.1002/bdra.23554

Mohammad, K. S., Chirgwin, J. M., & Guise, T. A. (2008). Assessing new bone formation in neonatal calvarial organ cultures. *Methods Mol Biol, 455*, 37-50. doi:10.1007/978-1-59745-104-8_3

Schriefer, J. L., Robling, A. G., Warden, S. J., Fournier, A. J., Mason, J. J., & Turner, C. H. (2005). A comparison of mechanical properties derived from multiple skeletal sites in mice. *J Biomech, 38*(3), 467-475. doi:10.1016/j.jbiomech.2004.04.020

Shearwin, K. E., Callen, B. P., & Egan, J. B. (2005). Transcriptional interference--a crash course. *Trends Genet, 21*(6), 339-345. doi:10.1016/j.tig.2005.04.009

Takahashi, N., Udagawa, N., Kobayashi, Y., & Suda, T. (2007). Generation of osteoclasts in vitro, and assay of osteoclast activity. *Methods Mol Med, 135*, 285-301. doi:10.1007/978-1-59745-401-8_18

Wang, B., Zheng, Y., Shi, H., Du, X., Zhang, Y., Wei, B., . . . Xu, X. (2017). Zfp462 deficiency causes anxiety-like behaviors with excessive self-grooming in mice. *Genes Brain Behav, 16*(2), 296-307. doi:10.1111/gbb.12339


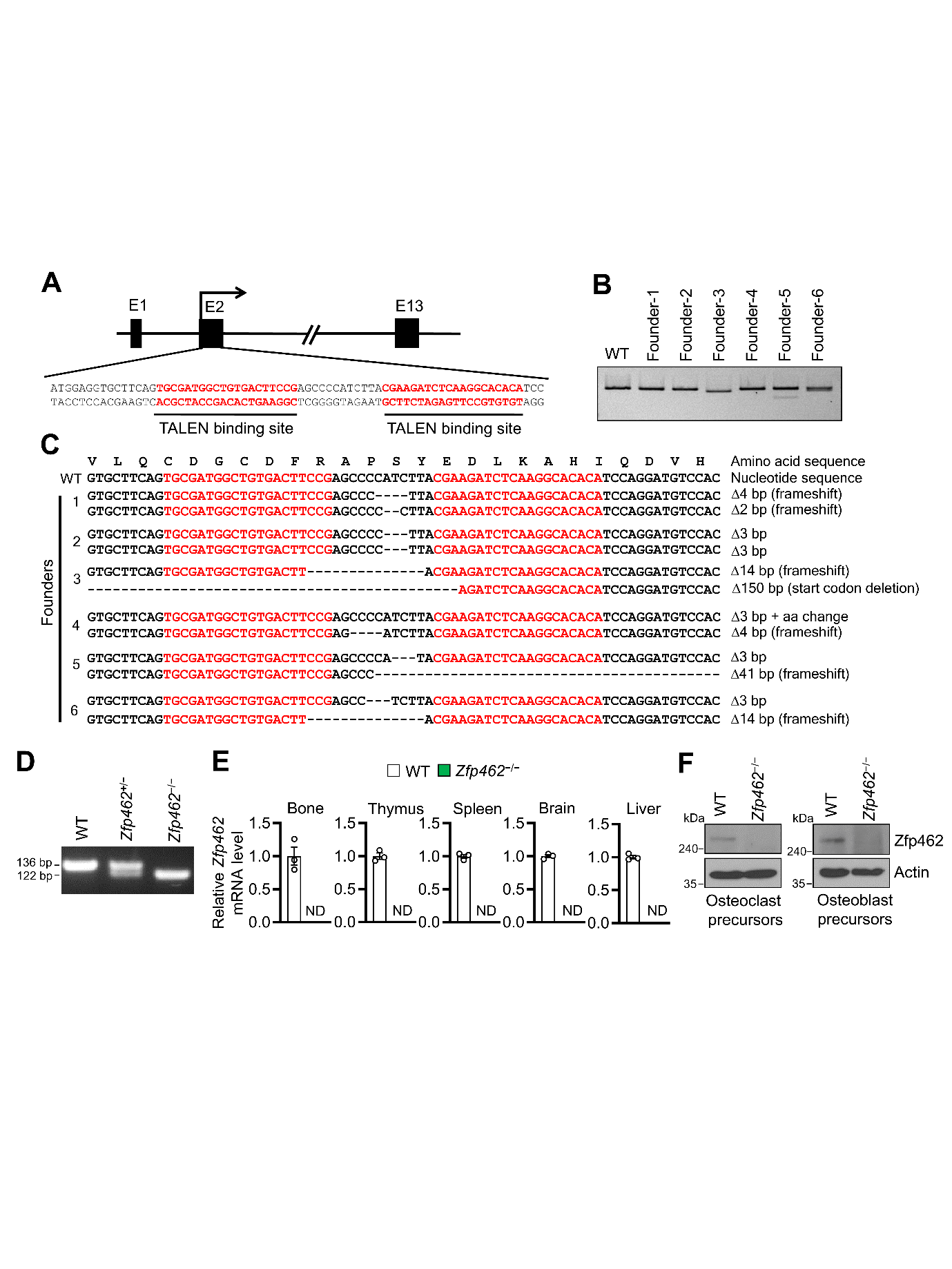


**Figure S1.** Generation of *Zfp462*-deleted mice using the TALEN system.

(A) Regions bound by *Zfp462*-TALEN within the murine *Zfp462* locus (red sequences). Black boxes indicate *Zfp462* locus exons. (B) PCR genotyping of *Zfp462* in tail samples from six *Zfp462* deletion mutant founder mice. Mutants were generated via intra-cytoplasmic injection of embryos with *Zfp462-*TALEN mRNA. (C) DNA sequence of *Zfp462* locus in the six *Zfp462* deletion mutant mice. Amino acid sequence is shown at the top. Dashes indicate deleted nucleotides. Deletion size (△) is shown to the right. The corresponding effect of mutation on the amino acid sequence is shown in brackets to the right. (D) Genotyping PCR analysis of WT, *Zfp462^+/-^*, and *Zfp462^−/−^* mice. Mutants were derived from Founder #3. Gene bands corresponding to WT *Zfp462* and *Zfp462* deletion mutant are 136 and 122 bp, respectively. (E) *Zfp*462 mRNA expression levels in organs from 4-week-old male WT and *Zfp462^−/−^* mice (*n* = 3/group). mRNA expression was normalized to *18S*. ND, not detected. (F) Western blot analysis of Zfp462 in osteoclast and osteoblast precursors derived from WT and *Zfp462^−/−^* mice. Actin served as a loading control. Data are shown as mean ± SEM.


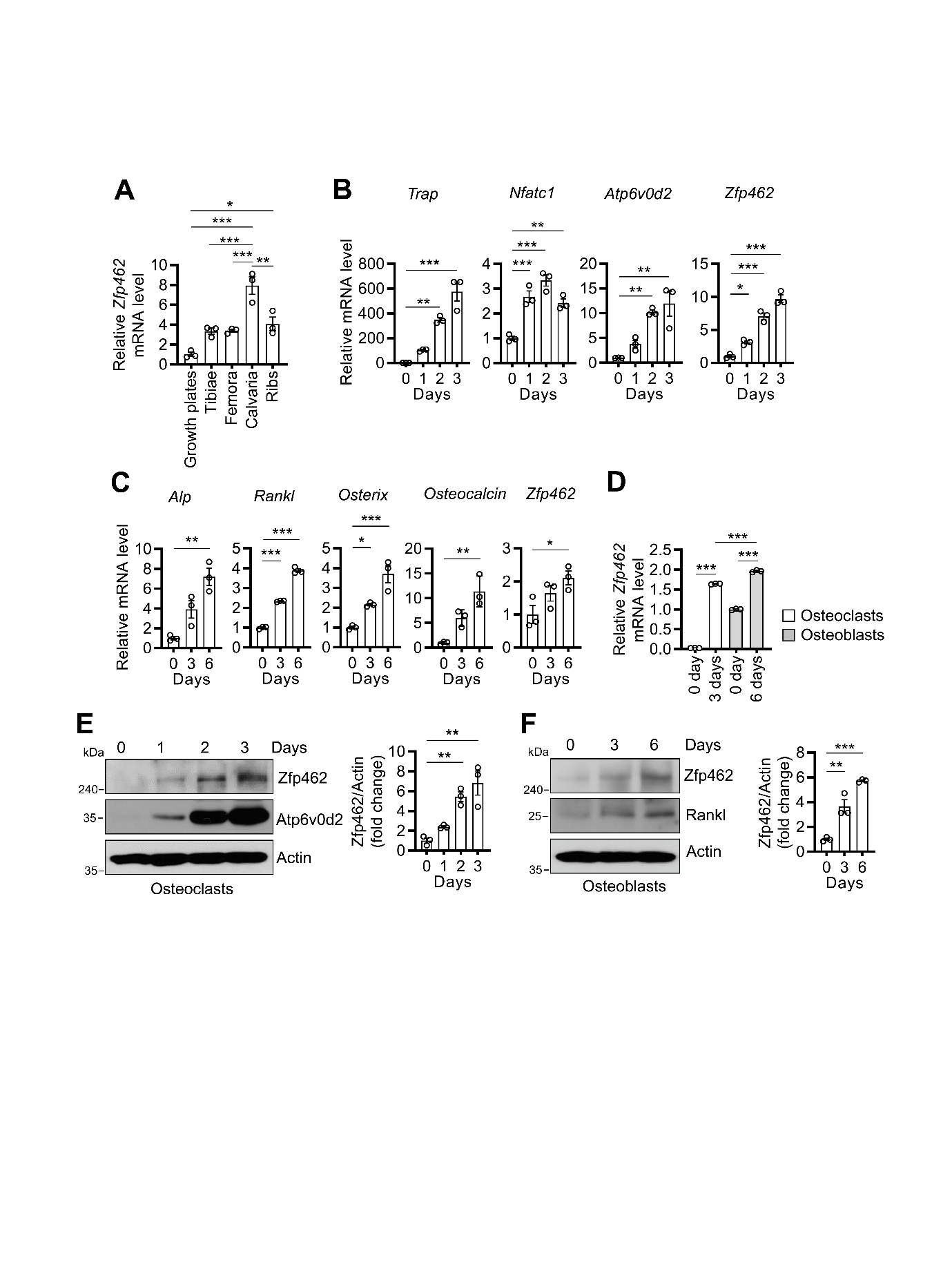


**Figure S2.** Zfp462 expression in various bones and during osteoblast and osteoclast differentiation.

(A) Zfp462 mRNA expression was analyzed by qRT-PCR in the indicated bone tissues isolated from 16-week-old male C57BL/6 mice, with growth plates obtained from femurs. Expression levels were normalized to *18S*. (B–D) Bone marrow macrophages (B, D) were incubated with 100 ng/mL RANKL and 30 ng/mL M-CSF for the indicated time points to generate osteoclasts. *Zfp462* and osteoclast markers *Trap*, *Nfac1*, and *Atp6v0d2* were analysed using qRT-PCR and normalized to *18S*. Calvarial osteoblast precursors (C, D) were differentiated into osteoblasts with osteogenic medium for the indicated times. *Zfp462* and osteoblast markers *Alp*, *Rankl*, *Osterix*, and *Osteocalcin* were analyzed using qRT-PCR and normalized to *18S*. (E and F) Osteoclasts (E) and osteoblasts (F) were differentiated as described in (B) and (C), respectively, and then subjected to western blots for the indicated factors. (Right) Band density of Zfp462. Actin served as a loading control. Data are shown as mean ± SEM of three experiments. Statistical analysis was conducted using one-way ANOVA (A–C, E, F) or two-way ANOVA (D) followed by Tukey’s test. **P* < 0.05, ***P* < 0.01, ****P* < 0.001.

**
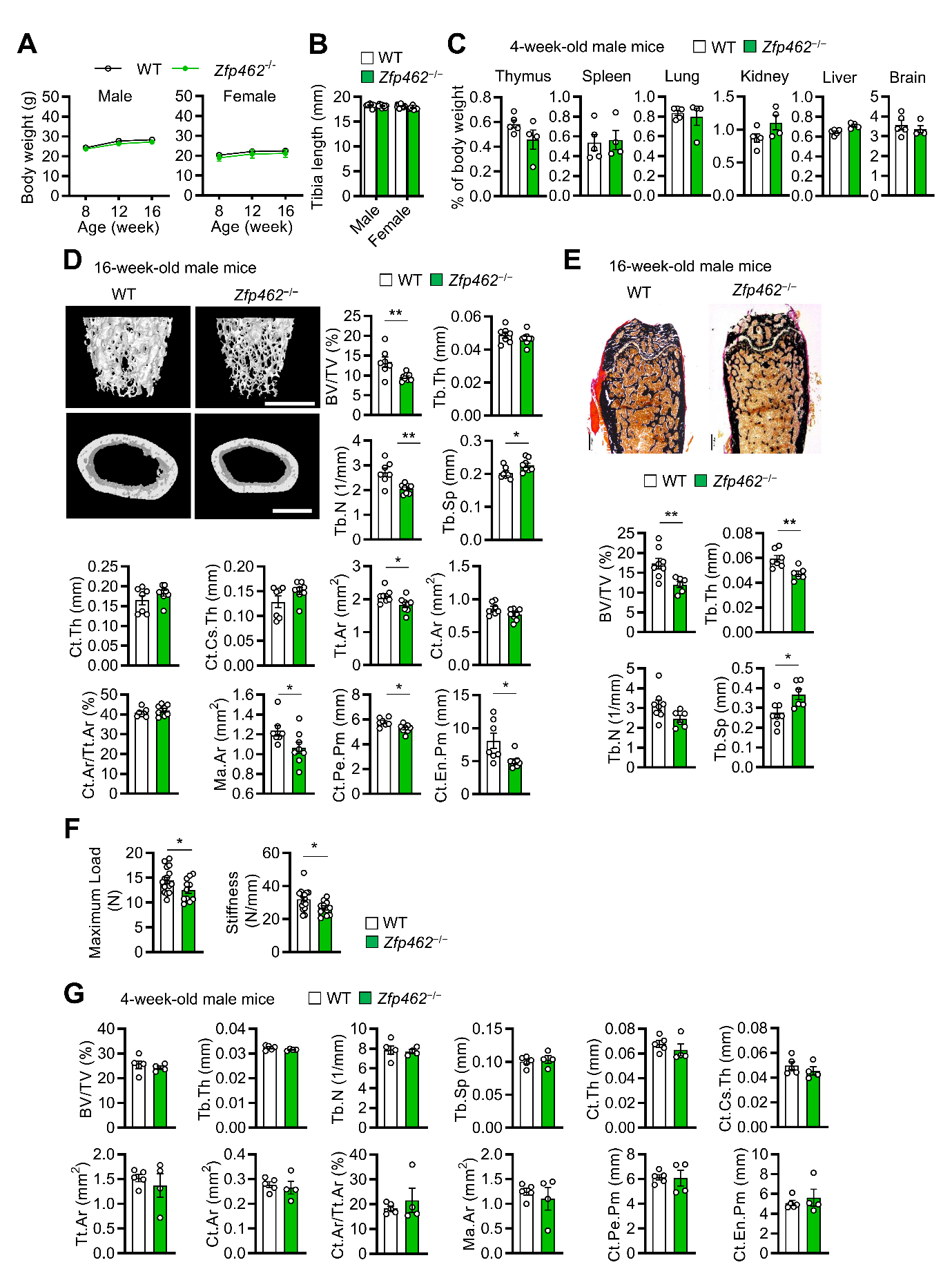
**

**Figure S3.** Organ weights and bone phenotype in male whole-body *Zfp462*-deficient mice at 4 and 16 weeks of age.

(A) Body weight of male (left) and female (right) *Zfp462^−/−^* mice (both *n* = 8) and their wild-type (WT) littermates (male *n* = 7; female *n* = 12). (B) Tibial length was measured in 16-week-old *Zfp462^−/−^* mice (both *n* = 10) and their WT littermates (male *n* = 9; female *n* = 15). (C) Relative weight of organs to body weight in 4-week-old male WT (*n* = 5) and *Zfp462^−/−^* (*n* = 4) mice. (D) Representative micro-CT images and quantification of femoral trabecular and cortical bone variables from 16-week-old male WT (*n* = 7) and *Zfp462^−/−^* (*n* = 8) mice. Scale bar, 1000 μm. BV/TV, trabecular bone volume per tissue volume; Tb.Th, trabecular thickness; Tb.N, trabecular number; Tb.Sp, trabecular spacing; Ct.Th, cortical thickness; Ct.Cs.Th, cortical cross-sectional thickness; Tt.Ar, cortical tissue area; Ct.Ar, cortical area; Ma.Ar, marrow area; Ct.Pe.Pm, cortical periosteal perimeter; Ct.En.Pm, cortical endosteal perimeter. (E) Representative Von Kossa-stained femur images from 16-week-old male WT and *Zfp462^−/−^* mice. Quantitative results are shown to the below (WT = 8 and *Zfp462^−/−^* = 6 for BV/TV, Tb.N, and Tb.Sp; and WT = 7 and *Zfp462^−/−^* = 6 for Tb.Th). (F) Maximum load (left) and stiffness (right) of tibiae from 16-week-old female WT (*n* = 16) and *Zfp462^−/−^* (*n* = 11) mice as measured via 3-point bending analysis. (G) Micro-CT of femurs from 4-week-old male WT (*n* = 5) and *Zfp462^−/−^* (*n* = 4) mice. Data are shown as mean ± SEM. Statistical significance was determined via Student’s unpaired two-tailed *t*-test. **P* < 0.05, ***P* < 0.01.


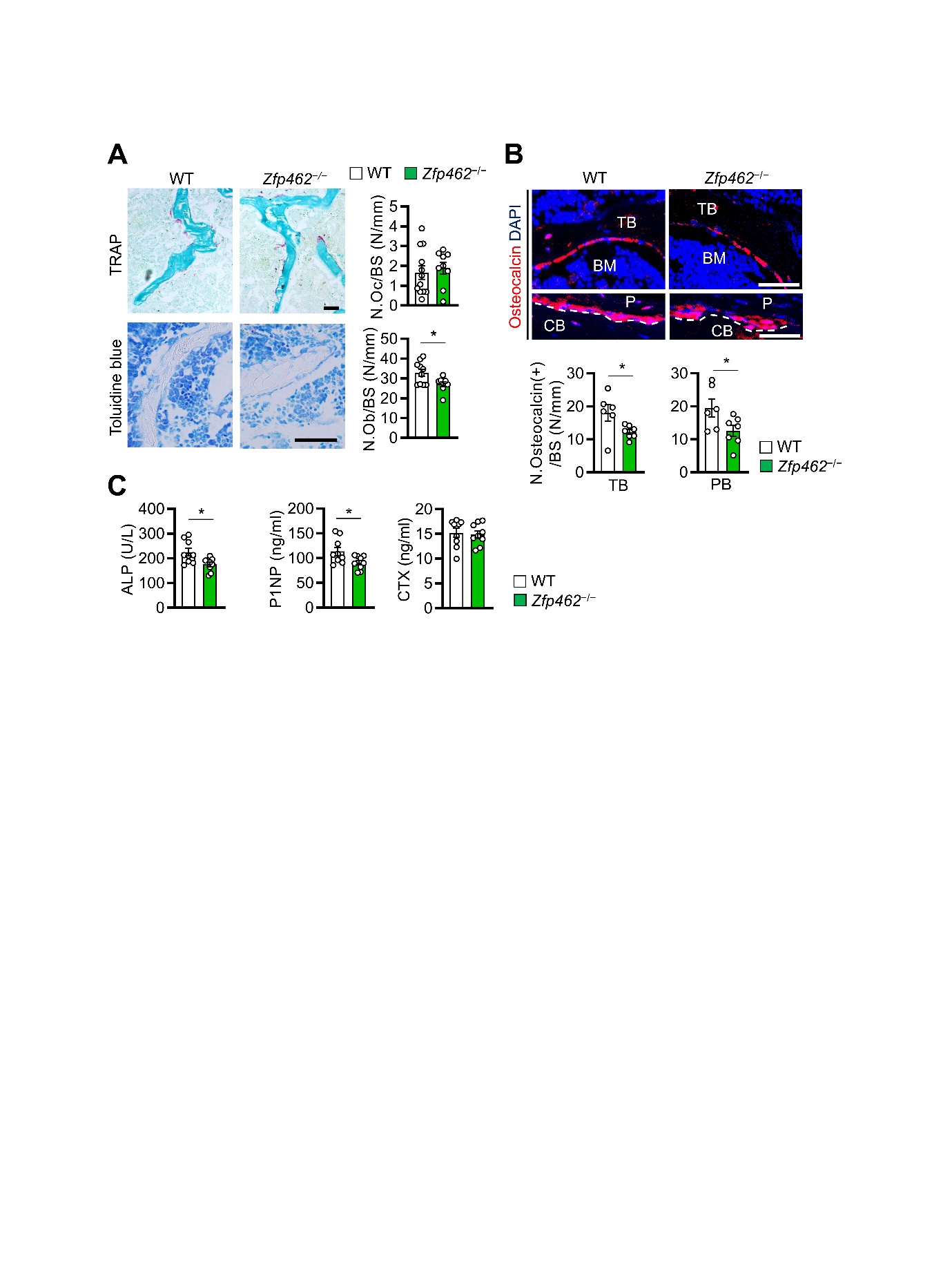


**Figure S4.** *Zfp462* deficiency decreases the number of osteoblasts, but not the number of osteoclasts.

Histological analysis of femurs from 16-week-old female *Zfp462^−/−^* mice and WT littermates. (A) Representative TRAP and toluidine-blue-stained femur images from WT (*n* = 12 for TRAP; and *n* = 10 for toluidine blue) and *Zfp462^−/−^* mice (*n* = 9 for TRAP; and *n* = 8 for toluidine blue). Scale bar, 50 μm. (Right) Osteoclast number (N.Oc/BS) and osteoblast number per bone surface (N.Ob/BS). (B) Representative osteocalcin-immunostained (red) and DAPI-stained (blue) femurs from WT (*n* = 6) and *Zfp462^−/−^* mice (*n* = 7). Scale bar, 50 μm. BM, bone marrow; TB, trabecular bone; CB, cortical bone; P, periosteal. (Below) Osteocalcin-positive cell numbers on trabecular bone (TB) and periosteal bone (PB) per bone surface. (C) Serum levels of alkaline phosphatase (ALP) activity, procollagen-1 N-terminal peptide (P1NP), and collagen type-I C-terminal telopeptide (CTX) in WT and *Zfp462^−/−^* mice (both *n* = 9), as measured using ELISA. Data are shown as mean ± SEM. Statistical significance was determined using Student’s unpaired two-tailed t-test. **P* < 0.05.

**
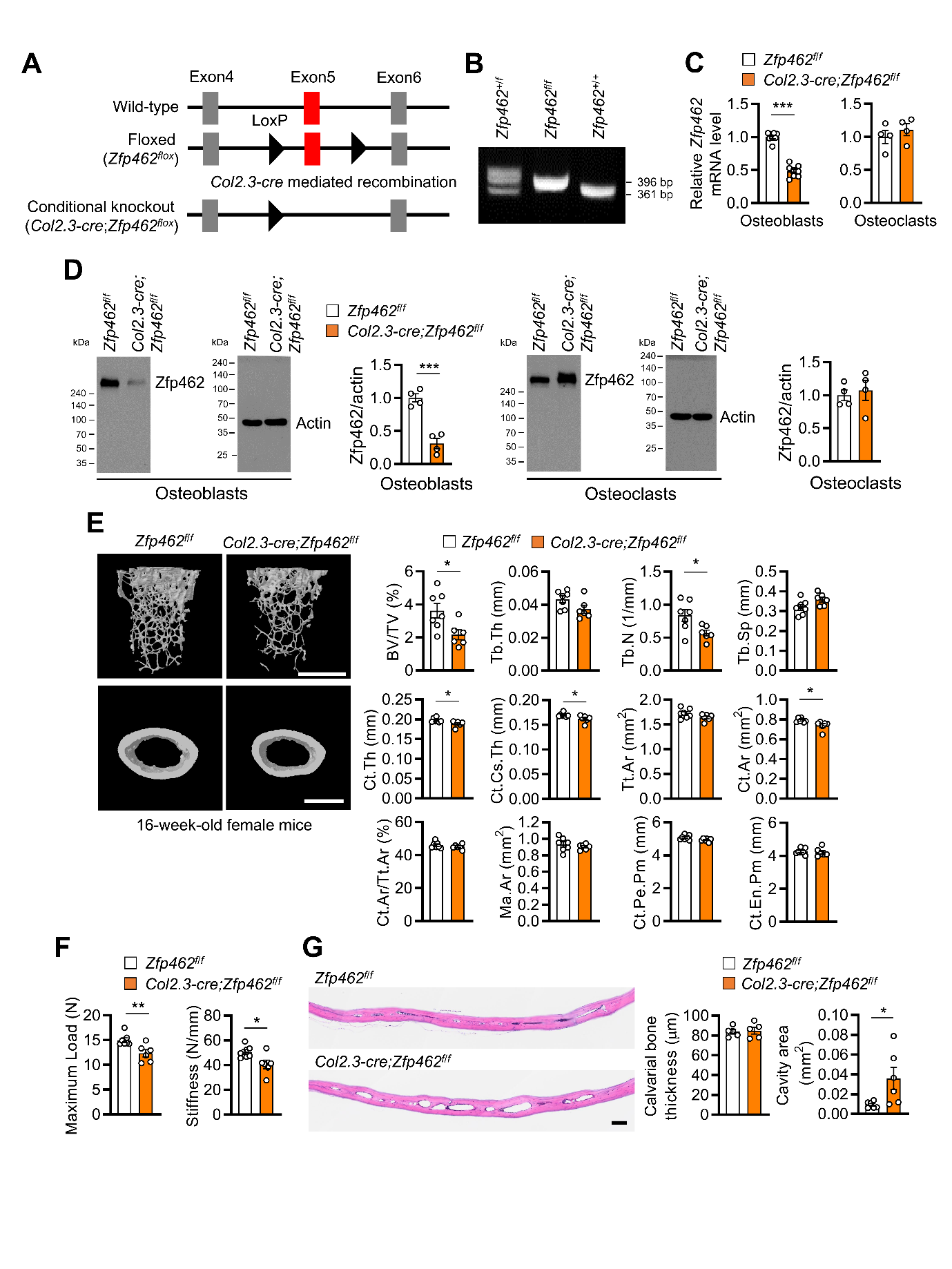
**

**Figure S5.** Generation of *Col2.3-cre*;*Zfp462^f/f^* mice and determination of bone mass and strength.

(A) Schematic depiction of WT, floxed Zfp462 (*Zfp462^flox^*), and conditional knockout (*Col2.3-cre*;*Zfp462^flox^*). *Zfp462* floxed (*Zfp462^f/f^*) mice were mated with *Col2.3-cre* transgenic mice to delete exon 5 of the *Zfp462* gene and generate conditional knockout *Col2.3-cre*;*Zfp462^f/f^* mice via Cre-mediated recombination. (B) Genotyping PCR analysis of *Zfp462^+/f^*, *Zfp462^f/f^*, and *Zfp462^+/+^* mice. Bands corresponding to *Zfp462^+/+^* and *Zfp462^f/f^* are 361 and 396 bp, respectively. (C and D) Osteoblast precursors and bone marrow macrophages from 16-week-old male *Zfp462^f/f^* and *Col2.3-cre*;*Zfp462^f/f^* mice were differentiated *in vitro* into osteoblasts and osteoclasts, respectively. (C) *Zfp462* mRNA expression in osteoblasts (*n* = 8 for both strains) and osteoclasts (*n* = 4 for both strains) was determined via qRT-PCR and normalized to *18S*. (D) Zfp462 protein expression in osteoblasts (*n* = 4 for both strains) and osteoclasts (*n* = 4 for both strains) was determined using western blot analysis. Actin served as a loading control. (Right) Band densities. (E) Micro-CT of femurs from 16-week-old female *Col2.3-cre*;*Zfp462^f/f^* mice (*n* = 6) and their *Zfp462^f/f^* littermates (*n* = 7). Scale bar, 1000 μm. BV/TV, trabecular bone volume per tissue volume; Tb.Th, trabecular thickness; Tb.N, trabecular number; Tb.Sp, trabecular spacing; Ct.Th, cortical thickness; Ct.Cs.Th, cortical cross-sectional thickness; Tt.Ar, cortical tissue area; Ct.Ar, cortical area; Ma.Ar, marrow area; Ct.Pe.Pm, cortical periosteal perimeter; and Ct.En.Pm, cortical endosteal perimeter. (F) Maximum load (left) and stiffness (right) of the tibiae of 16-week-old female *Col2.3-cre*;*Zfp462^f/f^* mice (*n* = 6) and *Zfp462^f/f^* littermates (*n* = 7), as measured using 3-point bending analysis. (G) Representative hematoxylin and eosin-stained calvaria images from 16-week-old female *Col2.3-cre;Zfp462^f/f^* mice (*n* = 6) and their *Zfp462^f/f^* littermates (*n* = 6). Scale bar, 100 μm. Quantitative results of calvarial thickness and cavity area from 16-week-old female *Zfp462^f/f^* and *Col2.3-cre;Zfp462^f/f^* mice (both *n* = 5 for thickness; both *n* = 6 for cavity area) are shown. Data are shown as mean ± SEM. Statistical significance was determined via Student’s unpaired two-tailed *t*-test. **P* < 0.05, ***P* < 0.01, ****P* < 0.001.

**
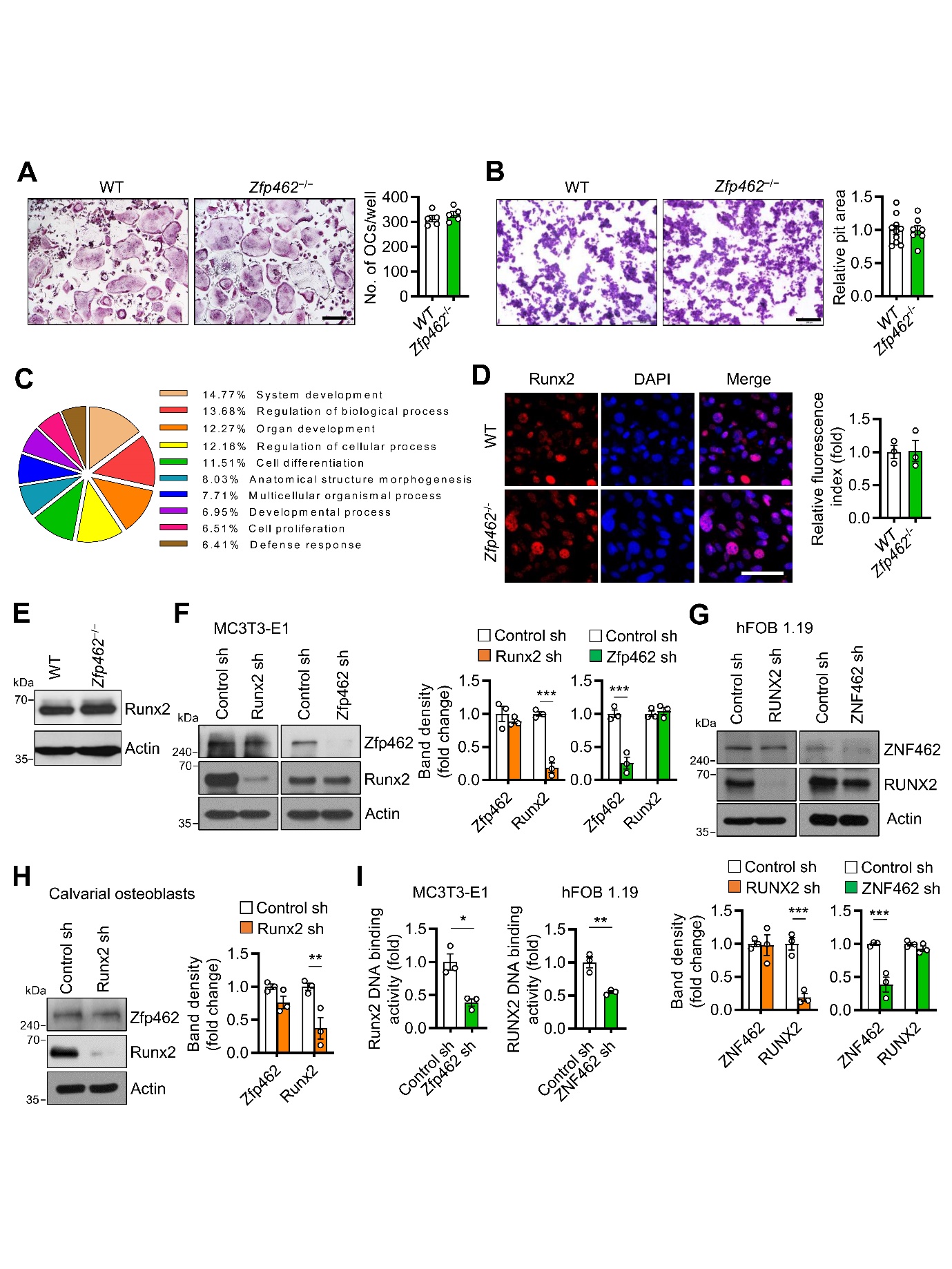
**

**Figure S6.** Effect of *Zfp462* deficiency on *in vitro* bone resorption and osteoblastic Runx2 expression.

(A) Formation of WT and *Zfp462^−/−^* osteoclasts from bone marrow macrophages. Macrophages were incubated with 100 ng/mL RANKL and 30 ng/mL M-CSF for 4 days and then stained with TRAP. Scale bar, 100 μm. TRAP-positive cells containing ≥ 3 nuclei and a complete actin ring were considered and counted as osteoclasts (right). (B) Bone resorption activity of WT (*n* = 10) and *Zfp462^−/−^* (*n* = 7) osteoclasts. Bone marrow macrophages were incubated with 100 ng/mL RANKL and 30 ng/mL M-CSF on dentin slices, rubbed off, and the dentin was then stained with hematoxylin and photographed (left). Scale bar, 100 μm. (Right) Pit areas were quantified using Image-Pro Plus program. (C) Gene ontology analysis of RNA sequencing data from calvarial osteoblasts of WT and *Zfp462^−/−^* mice. All genes affected by Zfp462 depletion were analysed using the DAVID online tool (<http://david.ncifcrf.gov>). (D and E) Effect of *Zfp462* deletion on Runx2 protein expression in osteoblasts. Calvarial osteoblast precursors from WT and *Zfp462^−/−^* mice were incubated in osteogenic medium. (D) After 3 days, osteoblasts were stained with anti-Runx2 (red) antibody and DAPI (blue). Scale bar, 100 μm. (Right) Total red fluorescence intensity values served as red fluorescence index. (E) After 4 days, osteoblasts were subjected to western blot analysis with an anti-Runx2 antibody. Actin served as a loading control. (F–H) Effect of silencing *Runx2* or *Zfp462/ZNF462* on *Runx2* and *Zfp462*/*ZNF462* expressions in murine MC3T3-E1 (F), human hFOB 1.19 (G), and calvarial osteoblast precursors (H). MC3T3-E1, hFOB 1.19, and calvarial osteoblast precursors were treated with *Runx2*/*RUNX2* or *Zfp462*/*ZNF462* sh-RNAs and then subjected to western blot analysis with the indicated antibodies. Band densities are shown on the right (F, H) or below (G). Actin served as a loading control. (I) Effect of silencing *Zfp462/ZNF462* on Runx2 DNA-binding activity. MC3T3-E1 (left) and hFOB 1.19 (right) osteoblastic cells were treated with *Zfp462/ZNF462* sh-RNA and incubated in osteogenic medium for 6 and 3 days, respectively, after which their nuclear fractions were subjected to Runx2 DNA-binding activity assays. Data are shown as mean ± SEM of three (D, F–I) or five (A) experiments. Statistical significance was determined using Student’s unpaired two-tailed *t*-test (I) or two-way ANOVA followed by Sidak’s post-hoc test (F–H). **P* < 0.05, ***P* < 0.01, ****P* < 0.001.

**
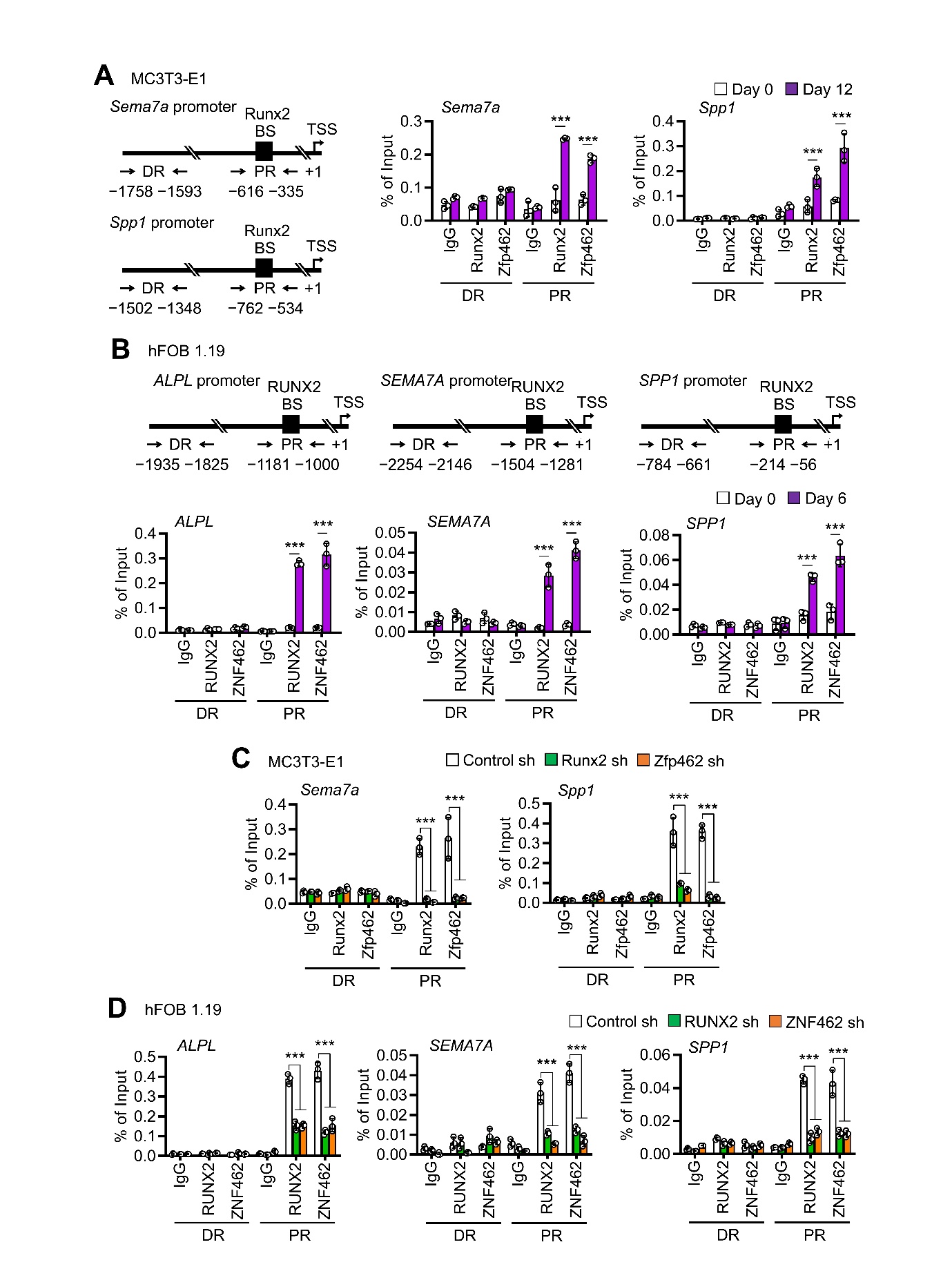
**

**Figure S7.** ChIP analysis of Runx2/RUNX2 and Zfp462/ZNF462 binding at ZNF462/Zfp462-target genes in osteoblasts.

(A) Zfp462 and Runx2 binding to *Sema7a* or *Spp1* locus in MC3T3-E1 osteoblasts, as determined by ChIP analyses using anti-Runx2 or anti-Zfp462 antibodies. (Left) Location of distal regions (DR) and proximal regions (PR) containing Runx2-binding sequences (BS) in the *Sema7a* and *Spp1* loci. Runx2-BS were identified using the patch public 1.0 program in TRANSFAC® Public 7.0 (http://gene-regulation.com/). (Right) MC3T3-E1 cells were induced to differentiate into osteoblasts in osteogenic medium for 12 days. ChIP analysis of *Sema7a* and *Spp1* loci was conducted before or after differentiation with ChIP primers that included Runx2-BS. (B) ZNF462 and RUNX2 binding to *ALPL*, *SEMA7A*, and *SPP1* loci in hFOB 1.19 osteoblasts, as determined by ChIP analyses using anti-RUNX2 or anti-ZNF462 antibodies. (Top) DR and PR locations of Runx2-BS in *ALPL*, *SEMA7A*, and *SPP1* loci. Runx2-BS were identified as described in (A). hFOB 1.19 cells were induced to differentiate into osteoblasts in osteogenic medium for 6 days. ChIP of *Sema7a* and *Spp1* loci was conducted before or after differentiation with ChIP primers that included Runx2-BS. (C and D) ChIP analysis of *Sema7a* and *Spp1* loci in MC3T3-E1 (C) and hFOB 1.19 cells (D) following Zfp462 or Runx2 silencing. Data are shown as mean ± SEM of three experiments. Statistical analysis was conducted using two-way ANOVA followed by Sidak’s post-hoc test. ****P* < 0.001.


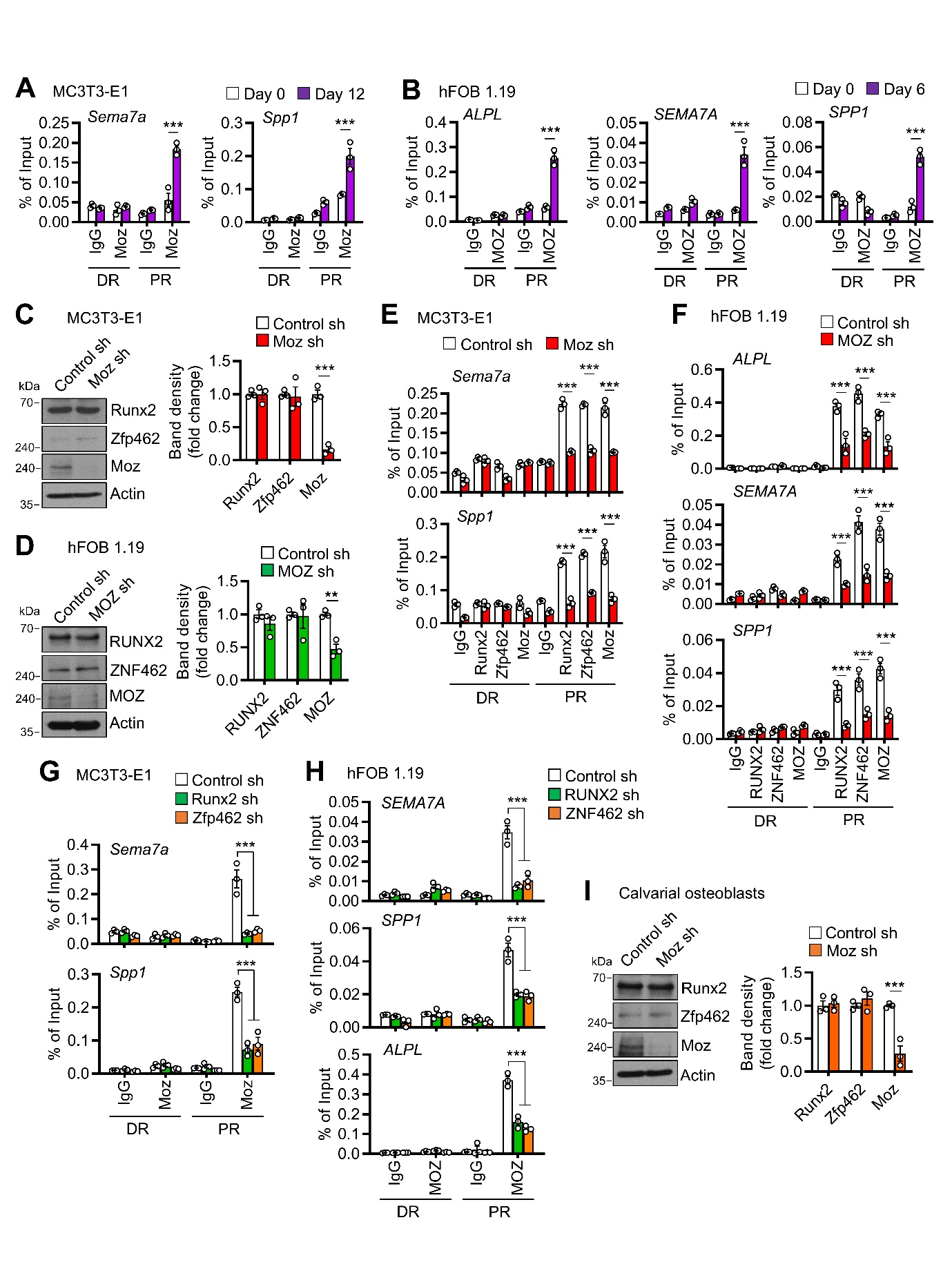


**Figure S8.** ChIP analysis of Runx2/RUNX2, Zfp462/ZNF462, and Moz/MOZ binding at ZNF462/Zfp462-target genes in osteoblasts.

(A) MC3T3-E1 cells were differentiated into osteoblasts with osteogenic medium for 12 days. Before and after differentiation, ChIP assays were performed at the distal regions (DR) and proximal regions (PR) of *Sema7a* and *Spp1* loci with anti-Moz antibody. (B) hFOB 1.19 cells were differentiated into osteoblasts with osteogenic medium for 6 days. Before and after osteogenesis, ChIP assays at *ALPL*, *SEMA7A*, and *SPP1* loci were performed using anti-MOZ antibody. (C and D) MC3T3-E1 (C) and hFOB 1.19 (D) cells were treated with *Moz/MOZ* sh-RNA and subjected to western blot analysis with the indicated antibodies. (Right) Band densities. Actin served as a loading control. (E and F) MC3T3-E1 (E) or hFOB 1.19 (F) cells were treated with *Moz/MOZ* sh-RNA as described in (C, D), and were then subjected to ChIP assays at the indicated loci with anti-Runx2/RUNX2, anti-Zfp462/ZNF462, or anti-Moz/MOZ antibodies. (G and H) MC3T3-E1 (G) or hFOB 1.19 (H) cells were treated with *Runx2/RUNX2* or *Zfp462/ZNF462* sh-RNAs as described in (C, D), and were then subjected to ChIP assays at the indicated loci with anti-Moz/MOZ antibody. (I) Western blot analysis of calvarial osteoblast precursors following Moz shRNA treatment with the indicated antibodies. (Right) Band densities. Actin served as a loading control. Data of three experiments are shown as mean ± SEM. Statistical analysis was conducted using two-way ANOVA followed by Sidak’s post-hoc test. ***P* < 0.01, ****P* < 0.001.


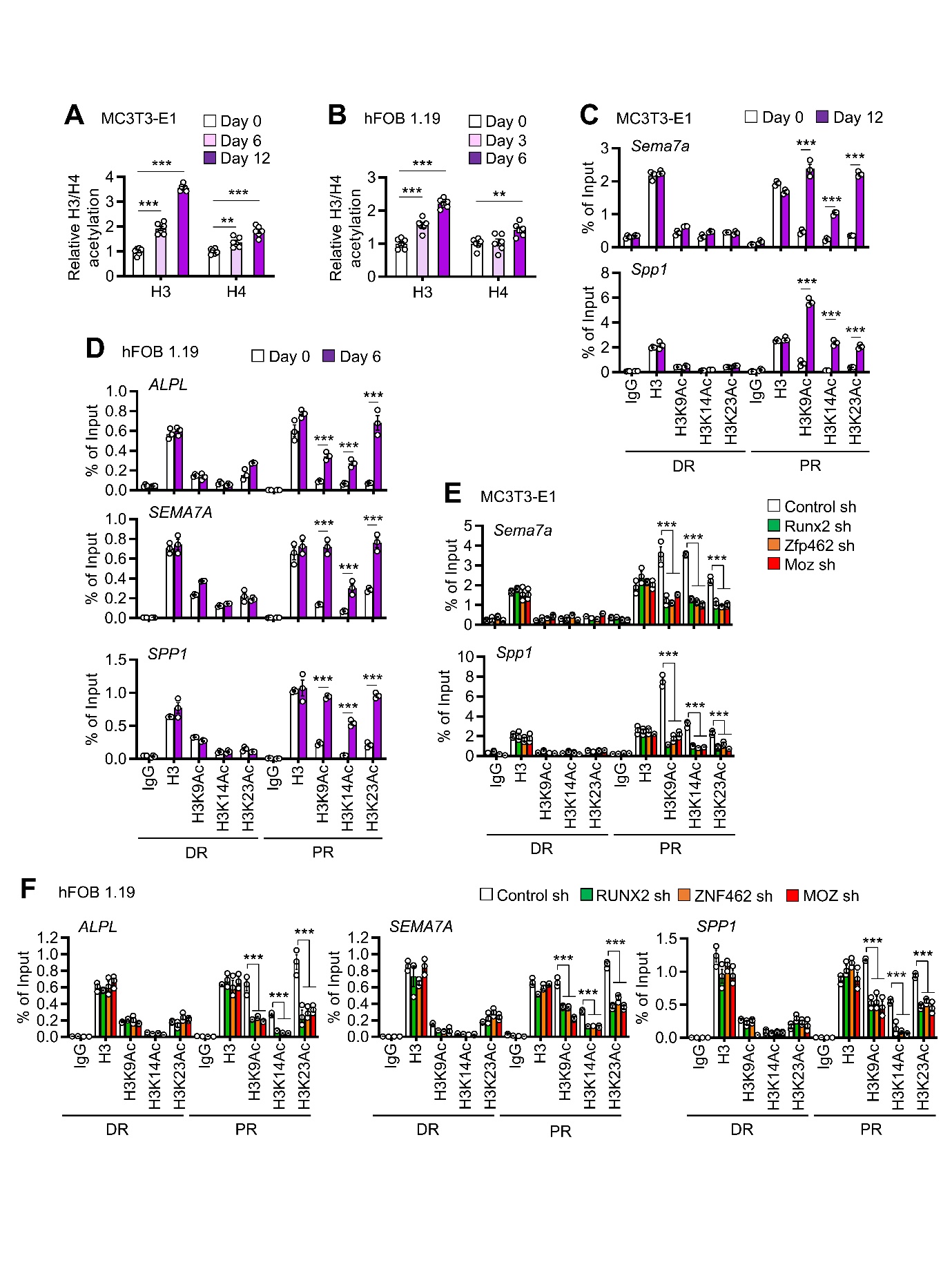


**Figure S9.** ChIP analysis of histone H3 acetylation at ZNF462/Zfp462-target genes in osteoblasts.

(A and B) MC3T3-E1 (A) and hFOB 1.19 (B) cells were cultured in osteogenic medium for the indicated durations and subjected to histone H3 and H4 acetylation assays. (C and D) MC3T3-E1 (C) and hFOB 1.19 (D) cells were cultured in osteogenic medium for 12 and 6 days, respectively. ChIP assays were performed at distal regions (DR) and proximal regions (PR) of *ALPL*, *SEMA7A*, and/or *SPP1* loci using the indicated antibodies. (E) MC3T3-E1 cells were treated with sh-RNAs against *Runx2*, *Zfp462*, or *Moz*, cultured in osteogenic medium for 12 days, and subjected to ChIP assays as described in (C). (F) hFOB 1.19 cells were treated with sh-RNAs against *RUNX2*, *ZNF462*, or *MOZ* and subjected to ChIP assays as described in (D). Data are shown as mean ± SEM of three experiments, except for (A) and (B) (*n* = 6). Statistical analysis was conducted using two-way ANOVA followed by Sidak’s post-hoc test. ***P* < 0.01, ****P* < 0.001.

**
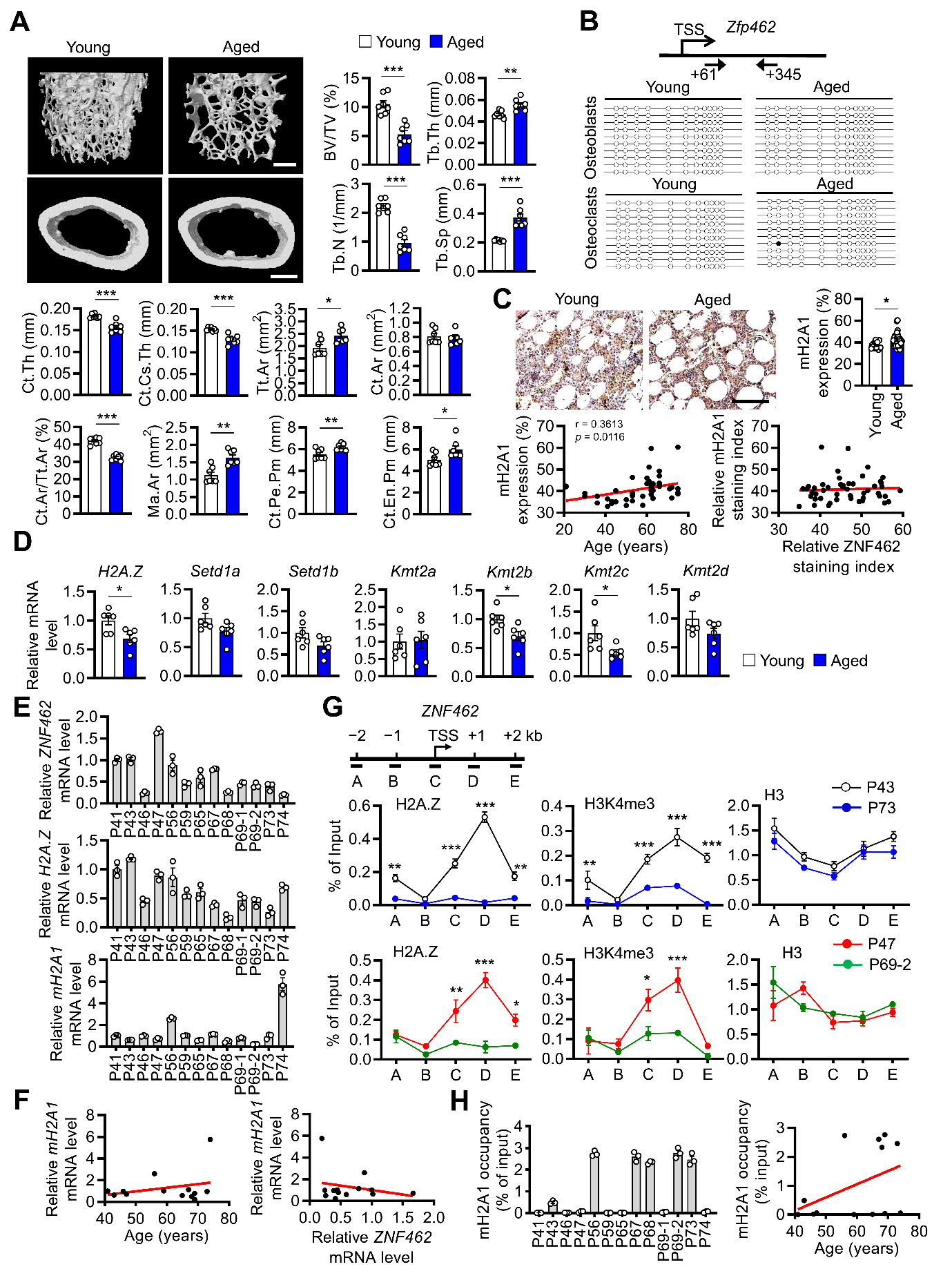
**

**Figure S10.** Histone variants in human bone marrow cells and their relationship with aging and *ZNF462* expression.

(A) Micro-CT analysis of femurs from 3-month-old (young) and 20-month-old (aged) male mice (both *n* = 7). Scale bar, 500 μm. BV/TV, trabecular bone volume per tissue volume; Tb.Th, trabecular thickness; Tb.N, trabecular number; Tb.Sp, trabecular spacing; Ct.Th, cortical thickness; Ct.Cs.Th, cortical cross-sectional thickness; Tt.Ar, cortical tissue area; Ct.Ar, cortical area; Ma.Ar, marrow area; Ct.Pe.Pm, cortical periosteal perimeter; and Ct.En.Pm, cortical endosteal perimeter. (B) Bisulfite sequencing analysis of *Zfp462* in young and aged male mice. (Upper) Schematic depiction showing the *Zfp462* gene region (+61 to +345), which included 11 CpG sites. TSS, transcription start site. (Lower) Representative sequencing data of osteoblasts and osteoclasts from young and aged mice. Each row represents an individual subclone. Methylated and unmethylated CpG sites are indicated by black and white circles, respectively. (C) Immunohistochemical staining of mH2A1 in human bone marrow tissue arrays from 24 subjects. (Top left) Representative cases. Scale bar, 100 μm. (Top right) mH2A1 expression in 6 young (<50-year-old) and 18 aged (≥50-year-old) subjects. (Bottom) Correlation between mH2A1 expression and either age or ZNF462 levels. (D) qRT–PCR analysis of *H2A.Z* and H3K4me3 methyltransferases (*Setd1a*, *Setd1b*, and *Kmt2a*–*d*) in tibial tissues from 12-week-old (young) and 20-month-old (aged) male mice. Expression levels were normalized to *18S*. (E–H) Human bone marrow stromal cells (BMSCs) obtained from 13 subjects without bone diseases were incubated in osteogenic medium for 6 days. (E) qRT-PCR of *ZNF462, H2A.Z*, and *mH2A1* in all individual patients, arranged from youngest to oldest. mRNA expression was normalized to *18S*. (F) Correlations between *mH2A1* levels and either age or ZNF462 levels. (G) Human BMSC-derived osteoblasts from two younger patients [aged 43 (P43) and 47 (P47) years] and two older patients [aged 69 (P69-2) and 73 (P73) years] were subjected to ChIP assays at the *ZNF462* locus with anti-H2A.Z, anti-H3K4me3, or anti-H3 antibodies. (Top) Approximate locations of five amplicons at the *ZNF462* locus. (Bottom) ChIP data of the four patients. (H) Human BMSC-derived osteoblasts were subjected to ChIP assays at *ZNF462* locus (+1 kb upstream of TSS; region D) with anti-mH2A1 antibody. (Left) Data of all individual patients, arranged from youngest to oldest. (Right) Correlation between mH2A1 levels at *ZNF462* locus and age. Data are shown as mean ± SEM. Statistical significance was determined using Student’s unpaired two-tailed t-test (A, C, D), two-way ANOVA followed by Sidak’s post-hoc test (G), or Pearson correlation analysis (C, F, H). **P* < 0.05, ***P* < 0.01, ****P* < 0.001.


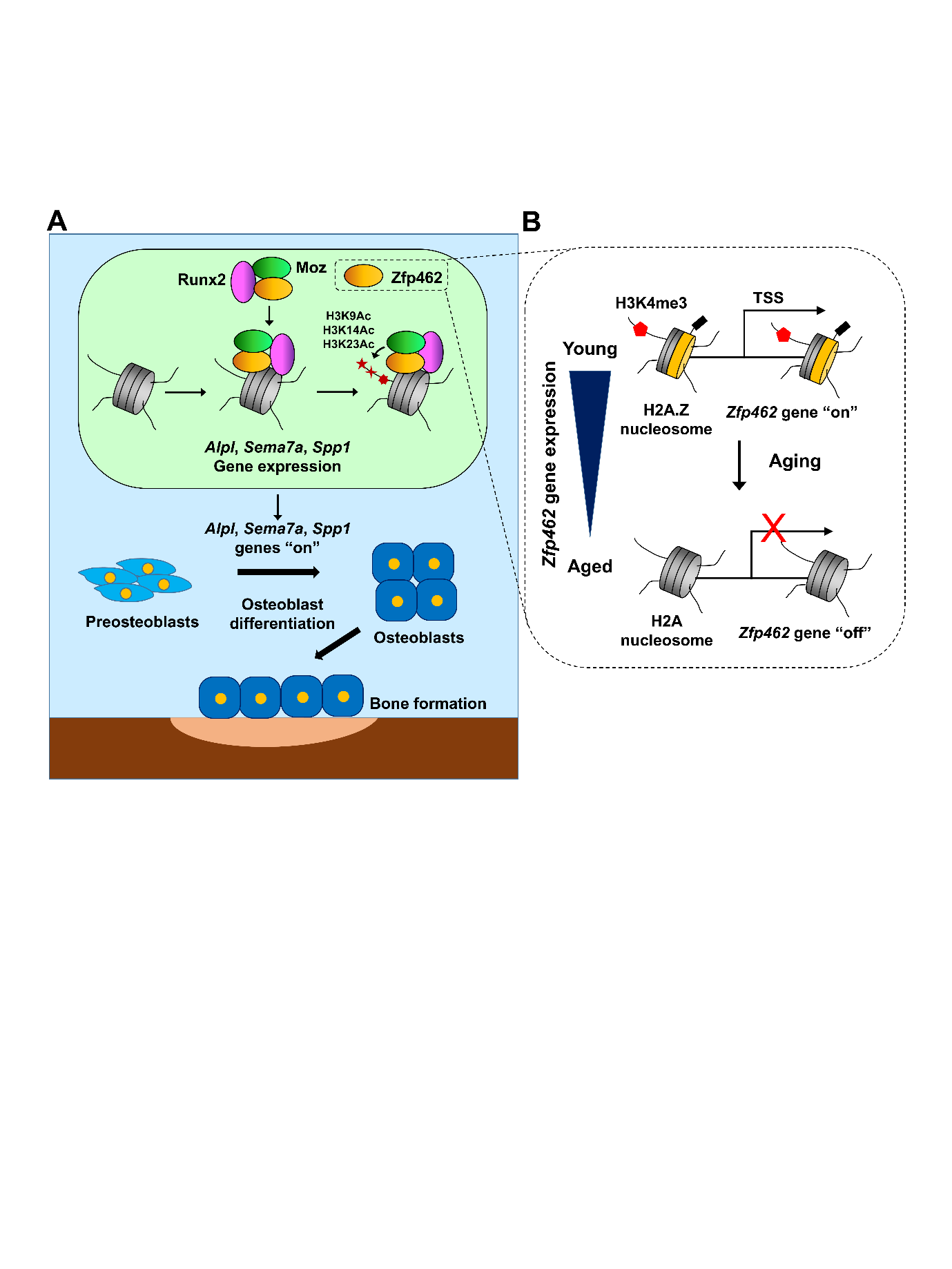


**Figure S11.** Proposed model of the mechanisms by which Zfp462-Moz-Runx2 complex modulates bone formation in an aging-dependent manner.

(A) The Zfp462-Moz-Runx2 complex binds to the promoter of Zfp462-target genes, which includes *Alpl*, *Sema7a*, and *Spp1*. Zfp462 facilitates the DNA binding ability of Runx2, which is a key regulator of osteoblast formation. Moz acetylates histone H3 lysine 9, 14, and 23, thereby increasing the expression of Zfp462-target genes and promoting osteoblast differentiation and bone formation. (B) During aging, nucleosome occupancy of the histone variant H2A.Z decreases at *Zfp462* locus. This reduces histone H3 lysine-4 tri-methylation and silences *Zfp462* gene. The resulting decrease in Zfp462 expression attenuates Runx2 transcriptional activity in osteoblasts and reduces bone formation.
